# Proteomic profiling identifies key coactivators utilized by mutant ERα proteins as potential new therapeutic targets

**DOI:** 10.1038/s41388-018-0284-2

**Published:** 2018-05-11

**Authors:** Leah A. Gates, Guowei Gu, Yue Chen, Aarti D. Rohira, Jonathan T. Lei, Ross A. Hamilton, Yang Yu, David M. Lonard, Jin Wang, Shu-Ping Wang, David G. Edwards, Philip F. Lavere, Jiangyong Shao, Ping Yi, Antrix Jain, Sung Yun Jung, Anna Malovannaya, Shunqiang Li, Jieya Shao, Robert G. Roeder, Matthew J. Ellis, Jun Qin, Suzanne A. W. Fuqua, Bert W. O’Malley, Charles E. Foulds

**Affiliations:** 1 Department of Molecular and Cellular Biology, Baylor College of Medicine, Houston, TX 77030 USA; 2 Laboratory of Chromatin Biology and Epigenetics, The Rockefeller University, New York, NY 10065 USA; 3 Lester and Sue Smith Breast Center, Baylor College of Medicine, Houston, TX 77030 USA; 4 Employee of Adrienne Helis Malvin Medical Research Foundation, New Orleans, LA 70130 USA; 5 Interdepartmental Graduate Program in Translational Biology and Molecular Medicine, Baylor College of Medicine, Houston, TX 77030 USA; 6 Department of Pharmacology, Baylor College of Medicine, Houston, TX 77030 USA; 7 Center for Drug Discovery, Baylor College of Medicine, Houston, TX 77030 USA; 8 Laboratory of Biochemistry and Molecular Biology, The Rockefeller University, New York, NY 10065 USA; 9 Verna and Marrs McLean Department of Biochemistry and Molecular Biology, Baylor College of Medicine, Houston, TX 77030 USA; 10 Division of Oncology, Department of Internal Medicine, Washington University School of Medicine, Saint Louis, MO 63110 USA; 11 Dan L. Duncan Cancer Center, Baylor College of Medicine, Houston, TX 77030 USA; 12 Center for Precision Environmental Health, Baylor College of Medicine, Houston, TX 77030 USA

## Abstract

Approximately 75% of breast cancers are estrogen receptor alpha (ERα)-positive and are treatable with endocrine therapies, but often patients develop lethal resistant disease. Frequent mutations (10–40%) in the ligand-binding domain (LBD) codons in the gene encoding ERα (*ESR1*) have been identified, resulting in ligand-independent, constitutively active receptors. In addition, *ESR1* chromosomal translocations can occur, resulting in fusion proteins that lack the LBD and are entirely unresponsive to all endocrine treatments. Thus, identifying coactivators that bind to these mutant ERα proteins may offer new therapeutic targets for endocrine-resistant cancer. To define coactivator candidate targets, a proteomics approach was performed profiling proteins recruited to the two most common ERα LBD mutants, Y537S and D538G, and an ESR1-YAP1 fusion protein. These mutants displayed enhanced coactivator interactions as compared to unliganded wild-type ERα. Inhibition of these coactivators decreased the ability of *ESR1* mutants to activate transcription and promote breast cancer growth in vitro and in vivo. Thus, we have identified specific coactivators that may be useful as targets for endocrine-resistant breast cancers.

## Introduction

Approximately 75% of breast cancers express estrogen receptor alpha (ERα) and new therapies are needed for the ~50% of ER-positive tumors that acquire endocrine resistance [1]. Current endocrine therapies include selective ERα modulators [2], aromatase inhibitors (AIs) [3], and selective ERα downregulators (SERDs) [4]. ERα plays a major role in the development of therapy-resistant tumors, and its activity is mediated by binding of 17-β estradiol (E2) to its ligand-binding domain (LBD) for the recruitment of steroid receptor coactivators (SRCs) and other coactivators for transcriptional activation of estrogen response element (ERE)-containing genes [5].

One mechanism for endocrine resistance is thought to be through acquired mutations in *ESR1* (gene encoding ERα). The first *ESR1* mutation (Y537N) was identified in a metastatic breast cancer patient and conferred ligand-independent transcriptional activity [6]. Since then, additional LBD point mutations have been identified that are expressed in a subset (10–40%) of metastatic tumors [7–14]. *ESR1* mutations appear to be acquired in response to AI treatment during metastatic progression [15] and are associated with poor survival [15–17]. The Y537S and D538G mutant receptors possess constitutive ERE-driven transcriptional activity [7–11]. These mutations lock helix 12 of the ERα LBD into an agonist-type conformation [7, 9, 18] and have higher affinities for the SRC-3 coactivator compared to unliganded wild-type (WT) ERα [14, 19].

*ESR1* chromosomal translocation events also occur in endocrine-resistant, metastatic breast cancer patients resulting in fusion proteins possessing the N terminus and DNA-binding domain (DBD) of ERα, but containing partners from various genes that replace the LBD [11, 20]. One such fusion, ESR1-YAP1, induced expression of ERE-containing target genes in a ligand-independent manner, and cannot be targeted with standard endocrine therapies since it lacks a LBD [11].

Understanding how ERα mutant proteins function is essential for the development of new therapeutics to treat endocrine-resistant tumors. To define co-regulators binding mutant ERα proteins as potential new targets, we profiled their recruitment to Y537S ERα, D538G ERα, and ESR1-YAP1 proteins bound to EREs using mass spectrometry (MS). We show that inhibition of the most-enhanced binding coactivators reduced ERE-driven transcription and *ESR1* mutant expressing breast cancer cell growth.

## RESULTS

### Identification of co-regulators recruited to ERα mutants

**Fig. 1:**
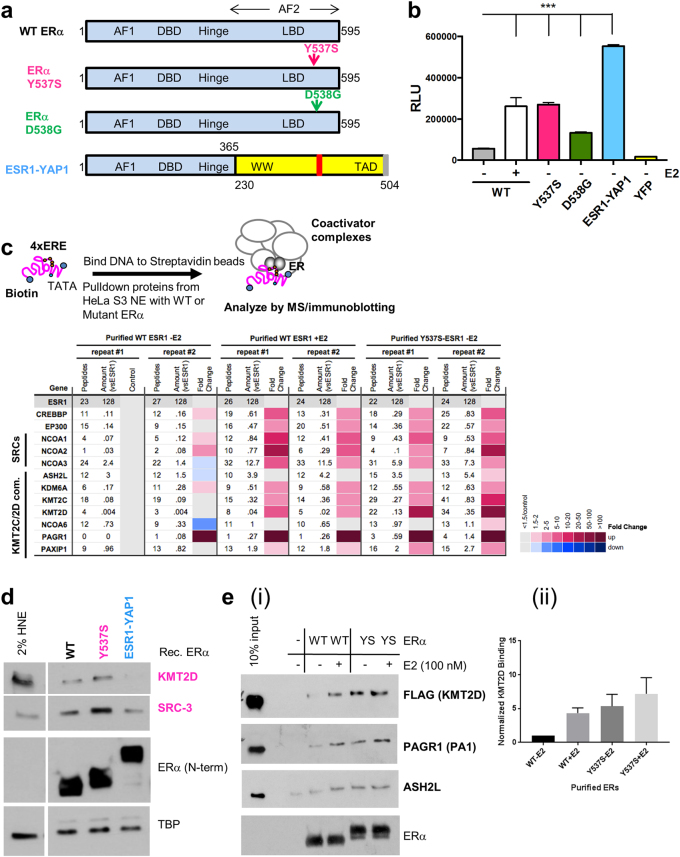
Proteomics of co-regulators recruited to the Y537S ERα LBD point mutant. **a** Schematic diagram of WT, Y537S, and D538G ERα point mutants (mutations indicated by arrows), and the ESR1-YAP1 fusion protein. Numbers refer to amino-acid residues in ERα and YAP1. *AF1* activation function 1, *DBD* DNA-binding domain. Hinge, region between DBD and ligand-binding domain (LBD); *AF2* Activation function 2, *WW* WW domain, *TAD* transcription activation domain. In ESR1-YAP1, blue represents ERα residues (1–365); yellow indicates YAP1 residues (230–504). **b** Mutant ERα proteins display E2-independent transcriptional activity. Vectors expressing YFP or YFP-tagged WT, Y537S, D538G, or ESR1-YAP1 proteins were co-expressed with an ERE-dependent luciferase reporter (pERE-E1b-luc) in HeLa cells grown in charcoal-stripped fetal bovine serum (FBS). Cells were then treated with/without 10 nM E2 for overnight. Luciferase activity (*RLU* relative light units) was assayed from whole-cell extracts of the cells transfected in triplicate. Data are represented as mean ± SEM (*n* = 3); ***p < 0.001. **c** MS data depicted as a heatmap for WT or Y537S ERα-dependent coactivators recruited to EREs. (Top) Schematic of ERE DNA pulldown assay using HeLa S3 NE as the source of co-regulators (modified from ref. [21]) and purified ERα proteins. (Bottom) MS data were analyzed from duplicate reactions using a label-free method and depicted as in ref. [21]. *Peptides* number of peptides detected; *amount (vsESR1)* amount normalized by sum of area under the curve for six N-terminal ESR1 peptides (see Supplementary Table 2). Fold change represents the ratio of amount detected normalized to unliganded WT ERα. Fold change cutoff used was ≥1.5. SRC-1 to -3 (gene symbols: NCOA1, NCOA2, NCOA3), p300 (gene symbol: EP300), CBP (gene symbol: CREBBP). **d** Immunoblotting validation of KMT2D and SRC-3 enrichment with purified Y537S ERα bound to EREs using independent DNA pulldown samples in the absence of E2. ERα protein binding was assayed by an N-terminal (N-term) antibody. TBP serves as a loading control. 2% input represents 2% of the starting HNE employed in the ERE DNA pulldown. **e** Y537S ERα and the KMT2D complex interact directly in an enhanced manner relative to unliganded WT ERα. ERE DNA pulldown assays were performed with purified ERα proteins and a purified KMT2D “fusion” complex [22], and then analyzed by immunoblotting. (i) Representative immunoblot probing for select KMT2D complex members and ERα. (ii) Quantification of KMT2D signal across three independent pulldown assays. Binding was quantified using Image J and normalized to ERα signal

Since Y537S, D538G, and ESR1-YAP1 ERα (Fig. 1a) promote E2-independent transcriptional activation of an ERE-dependent reporter (Fig. 1b), we tested whether this activation is through recruiting or repelling distinct co-regulators (coactivators and co-repressors). Using our 4xERE DNA pulldown assay to identify co-regulators recruited to ERα [21], we first utilized recombinant WT and Y537S ERα, along with HeLa S3 nuclear extract (HNE) to form complexes. Washed complexes were then subjected to label-free, quantitative MS and bound proteins were normalized by the amount of ESR1 N-terminal peptides bound (Fig. 1c, top; Supplementary Table 2).

Compared with unliganded WT ERα, a subset of coactivators were recruited in an enhanced manner to Y537S ERα (Fig. 1c, bottom). Namely, the histone H3 lysine 4 (H3K4) methyltransferase KMT2D complex displayed the greatest enrichment with Y537S ERα, along with SRC-1, -2, and -3, p300, CBP, and KMT2D’s paralog, KMT2C. Immunoblotting validated the enhanced KMT2D and SRC-3 recruitment (Fig. 1d).

As SRC-3 directly binds Y537S ERα [14, 19], we tested whether a KMT2D complex [22] would directly interact with Y537S ERα. Indeed, the binding of the KMT2D complex was enhanced with Y537S, along with E2-bound WT, compared to unliganded WT ERα (Fig. 1e). We found other potential coactivators with enhanced binding to Y537S ERα (Supplementary Figure 1a). We further validated PELP1 recruitment by immunoblotting, as an inhibitor disrupting this interaction is described [23] (Supplementary Figure 1b). We found very few co-repressors had reduced recruitment to Y537S ERα (Supplementary Figure 1c).

As purified D538G ERα failed to recruit SRC-3 despite binding EREs (data not shown), we resorted to using extracts from transfected 293T cells as sources of WT, Y537S, and D538G ERα proteins. We again observed enhanced recruitment of KMT2D and SRCs to Y537S compared to unliganded WT ERα (Supplementary Figure 2). The D538G mutant also recruited these coactivators, but three to four times less than that of Y537S. Additional potential co-regulators displayed enhanced binding to both ERα mutants. However, for subsequent functional characterization, we chose to focus on SRCs and the KMT2D complex.

### SRCs are critical for ERα LBD mutant activity and cell growth

**Fig. 2:**
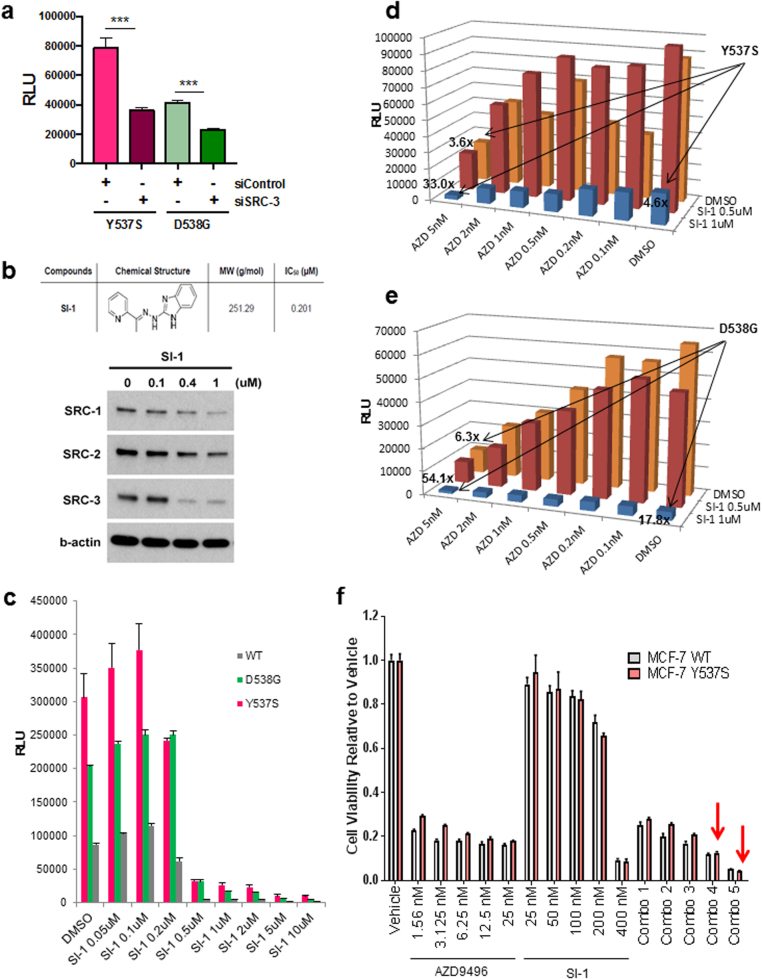
SRCs are key co-regulators of WT, Y537S, and D538G ERα transcriptional activity and are essential for breast cancer cell growth. **a** Knockdown of SRC-3 by co-transfection of HeLa cells with an siRNA targeting pool reduced transcriptional activity of LBD point mutant ERα proteins, as assayed by an ERE-luciferase reporter, in hormone-depleted media. Data are represented as mean ± SEM (*n* = 3); ***p < 0.001. *RLU* relative light units. **b** A “pan-SRC” inhibitor, SI-1, reduced all three SRC protein levels in MCF-7 breast cancer cells treated overnight, as assayed by immunoblotting. β-actin serves as a loading control. **c** SRC inhibitor SI-1 reduced WT, Y537S, and D538G ERα transcriptional activity at concentrations higher than the established IC_50_. Vectors expressing YFP-tagged WT, Y537S, and D538G ERα proteins were co-expressed with pERE-E1b-luc in HeLa cells, and then cells were treated with dimethyl sulphoxide (DMSO, vehicle control) or SI-1 overnight. Luciferase activity was measured as in Fig. 1b. Data are represented as mean ± SEM (*n* = 3). **d**,**e** Combination of an SRC inhibitor (SI-1) and an oral SERD (AZD9496) synergistically reduce Y537S (**d**) and D538G (**e**) mutant ERα transcriptional activity. Experiment was done as in (**c**), except that AZD9496 was added with or without SI-1 to co-transfected HeLa cells. Data are represented as mean (*n* = 3). **f** The oral SERD AZD9496 is more effective than the SRC inhibitor SI-1 in reducing cell viability of MCF-7 lines expressing WT or Y537S ERα. The lentiviral transduced MCF-7 stably expressing cell lines were treated with vehicle (DMSO) or different concentrations of AZD9496 or SI-1 as indicated. After 6 days of treatment, viability was assayed by a MTT assay. Data are represented as mean ± SEM (*n* = 3). Synergism was observed with combination (Combo) treatments 4 and 5 (12.5/200 nM AZD9496/SI-1; 25/400 nM AZD9496/SI-1) in Y537S ERα-expressing cells (shown as red arrows; CI values are shown in Supplementary Table 5)

We next tested the functional consequence of enhanced SRC-ERα mutant interactions on transcription. Knockdown of SRC-3 using published siRNAs [24] significantly reduced both Y537S and D538G ERα-mediated transcriptional activity on the ERE-Luc reporter (Fig. 2a). Treatment with a small molecule inhibitor (SMI), SI-1, which inhibits the activities of all three SRCs (IC_50_ = 0.2 μM) [25] and reduces SRC protein levels (Fig. 2b), severely reduced the transcriptional activities of WT and mutant ERα (Fig. 2c). Cell viability was minimally affected (Supplementary Figure 3a). We also tested whether the combination of an oral SERD and SI-1 would further reduce LBD mutant ERα transcriptional activity. We focused on AZD9496 [26] (AZD) as it: (1) reduced endogenous ERα protein, (2) was significantly more potent than ICI182,780 (ICI, fulvestrant) in reducing mutant ERα transcriptional activities (unlike another SERD GDC-0810 [27] (GDC; Supplementary Figure 3b-d), and (3) AZD was reported as more effective than ICI at inhibiting tumor growth promoted by Y537S ERα [14]. The combination of SI-1 and AZD synergistically reduced both Y537S and D538G activities on the ERE-luciferase (Luc) reporter (Fig. 2d, e, Supplementary Table 4).

We tested whether SRC inhibition would affect growth of stably expressing WT or Y537S ERα MCF-7 cell lines [28]. SI-1 at 400 nM reduced viability in both WT and Y537S ERα-expressing cells by 91% (Fig. 2f). When combinations of SI-1 with AZD or ICI were tested, a synergistic reduction in viability of Y537S ERα-expressing cells was found at the two highest combined doses or highest combined dose, respectively (Fig. 2f and Supplementary Figure 3e; Supplementary Tables 5 and 6). Thus, SI-1 combined with AZD was most effective in reducing both transcription and cell growth mediated by mutant ERα proteins.

### Inhibiting SRCs and mutant ERα most effectively reduces patient-derived xenograft tumor growth

**Fig. 3:**
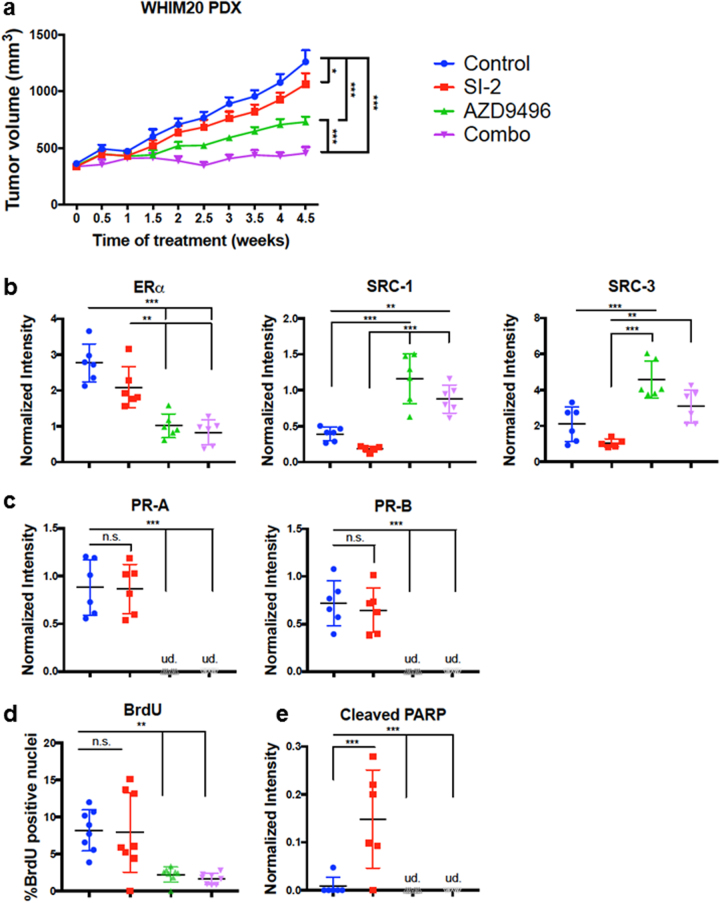
Inhibition of SRCs and ERα reduces tumor burden in a PDX model of Y537S ERα breast cancer. Significance was determined using one-way ANOVA and Tukey’s test to correct for multiple comparisons. *p < 0.05; **p < 0.01; ***p < 0.001. **a** Tumor volume (*n* = 8) is reduced with treatment of either SI-2 or AZD9496, and further reduced upon treatment with a combination of both inhibitors. Data are represented as mean ± SEM. **b–e** Quantification of tumor immunoblotting (*n* = 6/treatment) performed with Image J analysis relative to GAPDH expression or imaging data (*n* = 8) unless otherwise indicated. Data are represented as mean ± SD. *ud* undetected. Color legend is the same as in (**a**). **b** Quantification of ERα, SRC-1, and SRC-3 protein expression across tumors. By Dixon’s *Q* test, the tumor samples in the last lane probed for SRC-1 and SRC-3 (Supplementary Figure 4a) were outliers at 99% confidence and thus excluded from the Image J analysis. **c** The ERα target gene PR displays reduced expression with AZD9496 and combination treatment. **d** BrdU is decreased in the AZD9496 and combination therapy groups (*n* = 8 mice per group, with mean ± SD of three slides for each mouse counted). **e** Cleaved PARP is increased with SI-1 treatment and decreased with AZD9496 and combination therapies

We next tested the efficacy of an improved pan-SRC inhibitor (SI-2) [25], AZD, or the combination in a patient-derived xenograft (PDX) expressing Y537S ERα (WHIM 20 [11]) for tumor reduction (Fig. 3a). SI-2 was chosen instead of SI-1, given its reduced IC_50_ (3.4 nM) and ability to reduce ER^-^ tumor growth [25]. After tumors grew to 350 mm^3^, mice were randomized, E2 was withdrawn to mimic AI treatment, and mice were then treated with control vehicle, SI-2, AZD, or the combination. After 4.5 weeks, SI-2 alone significantly reduced tumor volume, AZD gave a larger reduction, but the combination gave the most significant reduction in tumor growth.

We next confirmed that the drugs indeed affected their intended targets and tested for effects on apoptosis and proliferation. First, as expected, AZD treatment significantly reduced ERα expression in tumor lysates. Unexpectedly, AZD treatment upregulated SRC expression (Fig. 3b, Supplementary Figure 4a), which may have relevance for patients receiving AZD monotherapy in clinical trials (NCT02248090/NCT03236974). Second, we tested the expression of a classical ER target gene, PR (Fig. 3c, Supplementary Figure 4a). While SI-2 did not affect PR expression, AZD clearly did. Third, we found that SI-2 increased an apoptosis marker (cleaved PARP protein), while AZD decreased proliferation as measured by BrdU incorporation (Fig. 3d, e, Supplementary Figure 4b). Finally, we did not observe any significant toxicity with any drug treatment after examining recipient mouse livers by histochemistry and measuring body weights (Supplementary Figures 4c-d). Thus, our PDX data support a potential new treatment regime for breast cancers bearing ESR1 LBD mutations, which is to combine a SRC inhibitor with an oral SERD.

### KMT2C/2D are novel coactivators for Y537S ERα

**Fig. 4:**
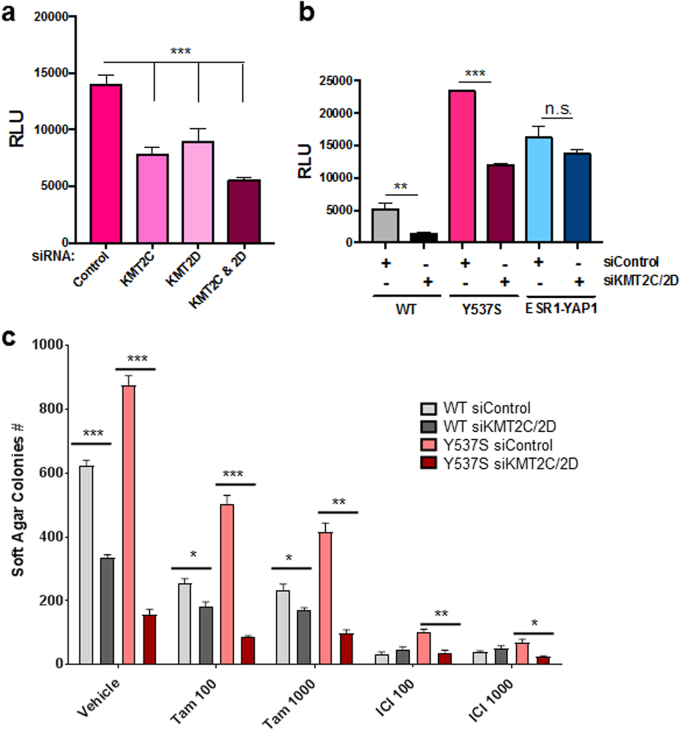
Knockdown of KMT2C/2D reduces WT and Y537S ERα transcriptional activity and breast cancer cell growth. **a** HeLa cells grown in phenol red-free, charcoal-stripped media were co-transfected with pERE-E1b-luc, YFP-tagged Y537S ERα, and different siRNAs (25 nM each for 50 nM total). Cell lysates were assayed for luciferase activity (RLU). Data are represented as mean ± SEM (*n* = 3); ***p < 0.001. NC#1 siRNA served as the negative control for KMT2D targeting siRNA, while Control-A siRNA pool served as the negative control for KMT2C targeting siRNA pool. **b** Transfection of siRNAs targeting KMT2C and KMT2D reduced expression of the pERE-E1b-luc reporter, as compared to non-targeting siRNAs (siControl), in HeLa cells co-transfected with YFP-tagged WT or Y537S ERα vectors, but not an YFP-tagged ESR1-YAP1 fusion. Luciferase activity was measured as in Fig. 1b. Data are represented as mean ± SEM (*n* = 3); **p < 0.01; ***p < 0.001. **c** Knockdown of KMT2C and KMT2D in lentiviral transduced stably expressing WT or Y537S MCF-7 cells results in reduced anchorage-independent growth in soft agar and confers sensitivity to anti-estrogens. siRNAs (same as above) were transfected into the two cell lines at a final concentration of 100 nM (50 nM each), and then re-plated in 24 well plates 24 h later. After 1 week with either vehicle or anti-estrogen (100 or 1000 nM 4-hydroxytamoxifen (Tam) or ICI) treatment, colonies formed in soft agar were counted and quantified. Data are represented as mean ± SEM (*n* = 4); *p < 0.05; **p < 0.01; ***p < 0.001

From above, we found that the KMT2C/2D complexes were preferentially enriched with Y537S ERα (Fig. 1c, Supplementary Figure 2a). To determine the functional role of KMT2C/2D, we tested whether their depletion would affect Y537S ERα-mediated reporter expression. Knocking down KMT2C, KMT2D, or both together reduced Y537S ERα transcriptional activity (Fig. 4a). Upon double KMT2C/2D knockdown, WT ERα transcriptional activity was also reduced (Fig. 4b). However, ESR1-YAP1 transcriptional activity was not affected, ruling out a general transcriptional effect.

We additionally found that KMT2C/2D knockdown reduced anchorage-independent growth of WT, Y537S, and D538G ERα-expressing cells (Supplementary Figure 5), with significant differences observed between WT and mutant ERα data. More importantly, knockdown of KMT2C/2D significantly sensitized the partially resistant Y537S ERα cells to anti-estrogens currently given in the clinic (Fig. 4c). Overall, our data reveal an important functional role for KMT2C/2D with Y537S ERα in transcription and cell growth.

### SRCs and KMT2D are crucial for growth of inducible LBD mutant ERα cells

**Fig. 5:**
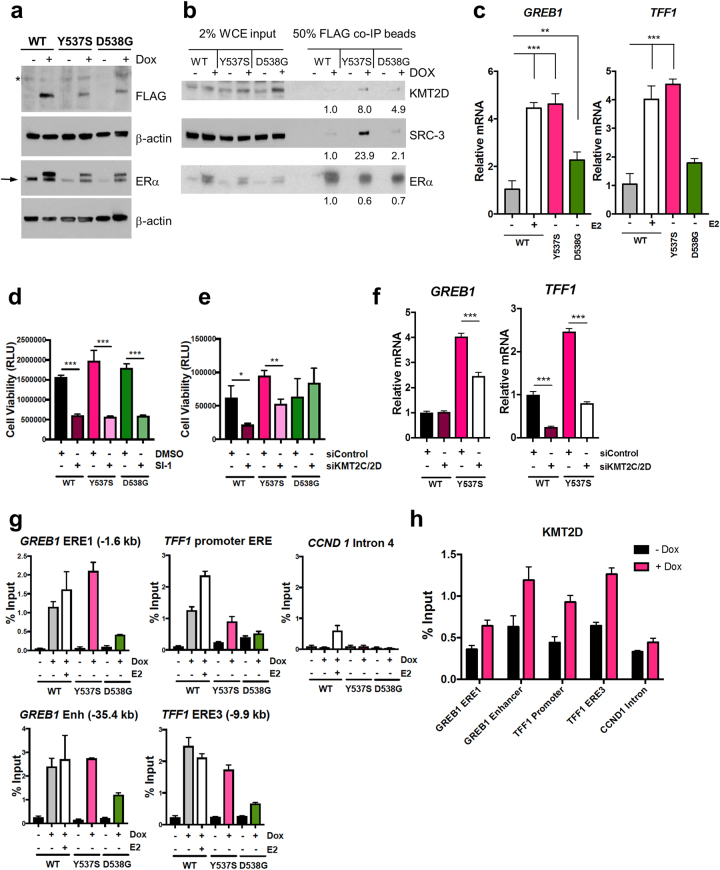
SRCs and KMT2C/2D promote cell growth and transcription of ERE-containing target genes in conditionally expressed (Dox-inducible) WT and mutant ERα MCF-7 cell lines. Prior to all assays, cell lines were grown in charcoal-stripped, phenol red-free media with/without Dox for at least 2 days. **a** Confirmation of FLAG-tagged WT, Y537S, and D538G ERα Dox-inducible expression in MCF-7 cells transduced with specific lentiviruses. Immunoblotting was performed on whole-cell extracts with FLAG, ERα (HC-20 antibody), or β-actin (loading control) antibodies. *, Nonspecific band. →, Position of endogenous ERα. **b** KMT2D and SRC-3 co-immunoprecipitate with mutant ERα. Whole-cell extracts from Dox-inducible MCF-7 cells were subject to immunoprecipitation of FLAG, followed by immunoblotting. For analysis, intensities were normalized for different precipitated ERα levels by Image J. **c** Dox-inducible expression of Y537S and D538G ERα in MCF-7 cells results in activation of two canonical ERα target genes independent of E2. Cell lines were treated with Dox and the WT ERα line was treated (±) 10 nM E2 overnight, followed by RNA isolation. Relative levels of *GREB1* or *TFF1* mRNAs were determined by RT-qPCR with *ESR1* mRNA as the normalizer. Data are represented as mean ± SEM (*n* = 3); **p < 0.01; ***p < 0.001. **d**, **e** Cell viability (measured by Cell Titer Glo as RLU) of Dox-inducible WT, Y537S, and D538G ERα expressing MCF-7 lines (induced as above). *p < 0.05; **p < 0.01; ***p < 0.001. **d** Cell viability is reduced in all three cell lines upon treatment with SRC inhibitor SI-1 (1 μM) after 3 days' exposure. DMSO served as the vehicle control. Data are represented as mean ± SEM (*n* = 3). **e** Cell viability of Dox-inducible WT and Y537S ERα-expressing MCF-7 lines, but not the D538G-expressing ERα line, is reduced upon knockdown of KMT2C and KMT2D after 3 days exposure to 100 nM total siRNA (50 nM each). Data are represented as mean ± SEM (*n* = 3). **f** Knockdown of KMT2C and KMT2D reduces Y537S ERα-enhanced expression of *GREB1* and *TFF1*. Cells were transfected with siRNAs (100 nM total, 50 nM each). Relative levels of *GREB1* or *TFF1* mRNAs were determined by RT-qPCR using *ACTB* mRNA as the normalizer. Data are represented as mean ± SEM (*n* = 3); *p < 0.05; **p < 0.01; ***, *p* < 0.001. **g** Dox-induced FLAG-tagged WT, Y537S, and D538G ERα proteins occupy EREs of *GREB1* and *TFF1* genes in MCF-7 cells, implying direct transcriptional regulation. In contrast, ERα proteins minimally occupy a negative control region from intron 4 of the *CCND1* gene, which lacks endogenous ERα binding [36]. Where indicated, WT ERα cells were treated with 100 nM E2 for 45 min. ChIP assays employed an antibody against FLAG to IP the FLAG-tagged ERα proteins and associated DNA. Isolated DNA was assayed by ChIP-qPCR. Representative data were plotted relative to percentage of starting input chromatin and are represented as mean of triplicate qPCR reactions ± SEM. Supplementary Figure 7d shows a repeated ChIP assay. **h** KMT2D occupies EREs of *GREB1* and *TFF1* genes in a Dox-dependent manner correlating with increased Y537S ERα occupancy. ChIP-qPCR was performed using an antibody to KMT2D. Representative data were plotted as above and the *CCND1* gene intron 4 served as a negative control region. Supplementary Figure 7e shows a repeated ChIP assay

As our above cell lines stably overexpressed LBD ERα mutants [28], we created MCF-7 cell lines supporting conditional (doxycycline (Dox)-inducible) expression of FLAG-tagged WT, Y537S, or D538G ERα. The FLAG tag did not impair the transcriptional activities of ERα proteins (Supplementary Figure 6). Dox addition to cells grown in charcoal-stripped media induced expression of these ERα proteins, with Y537S and D538G ERα accumulating to a similar extent but less than WT (Fig. 5a). We next performed co-immunoprecipitations to test whether SRC-3 and KMT2D displayed enhanced association with inducible mutant ERα proteins under hormone-depleted conditions. We found greatest KMT2D and SRC-3 association with the Y537S mutant, with less recruited to D538G ERα (Fig. 5b). Importantly, the Dox-inducible mutant ERα proteins activated endogenous ERα target genes (*GREB1* and *TFF1*) in a hormone-independent manner and D538G ERα had a weaker effect than Y537S, in accordance with other models [7, 9, 29–31] (Fig. 5c and Supplementary Figure 7a). We next asked whether ablation of these key co-regulators would inhibit the viability of these cells. Consistently, SI-1 significantly reduced the viability of all ERα-expressing cells (Fig. 5d), while knockdown of KMT2C/2D selectively affected WT and Y537S ERα-expressing cells (Fig. 5e).

### Knockdown of KMT2C/2D modulates Y537S ERα direct target gene expression

We next tested the effect of KMT2C/2D depletion on *GREB1* and *TFF1* gene expression in our Dox-inducible MCF-7 cells. KMT2C/2D knockdown reduced Y537S ERα-mediated transcription of both genes (Fig. 5f and Supplementary Figure 7b). In WT ERα cells, the loss of KMT2C/2D also reduced *TFF1* mRNA, but *GREB1* or *ESR1* were unaffected. We extended our analysis to 10 total ERα target genes by depleting only KMT2D using a validated siRNA [32], as KMT2D had greater recruitment than KMT2C to Y537S ERα (Fig. 1c). We observed that Y537S mutant expression regulates select ERα targets, a subset of which is reduced by KMT2D depletion (Supplementary Figure 7c).

Chromatin immunoprecipitation (ChIP) was used to examine whether the ERα mutants bound EREs [33–35] located upstream of the *GREB1* and *TFF1* gene transcription start sites (TSS) in a Dox-dependent manner (Fig. 5g and Supplementary Figure 7d). All ERα proteins displayed Dox-dependent enrichment on EREs upstream of the *GREB1* and *TFF1* TSS, but not on a negative control region [36]. Thus, the binding of the LBD mutant ERα proteins to multiple EREs suggests direct regulatory roles in regulation of these genes.

### Chromatin occupancy of Y537S ERα and KMT2D are positively correlated

As SRC-3 and p300 are co-localized with Y537S ERα on chromatin [29], we next tested whether KMT2D chromatin occupancy correlated with Y537S ERα. We performed ChIP using a validated KMT2D antibody [37, 38] (Fig. 5h and Supplementary Figure 7e). KMT2D occupancy increased in Y537S ERα cells in a Dox-dependent manner for all EREs assayed, but not for the control region, without increased KMT2D protein expression (Supplementary Figure 7f). Thus, Dox-induced occupancy of EREs by Y537S ERα is correlated with increased recruitment of KMT2D and enhanced transcription of *GREB1* and *TFF1*.

### Co-regulators recruited to the ESR1-YAP1 fusion

**Fig. 6:**
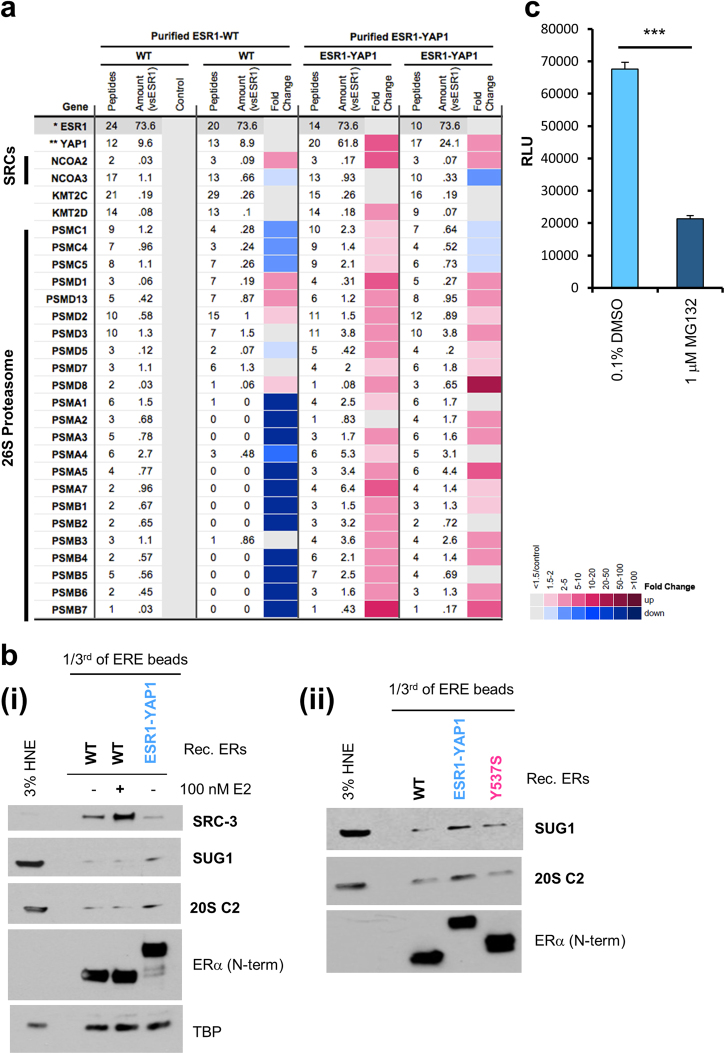
The ESR1-YAP1 fusion protein recruits the 26S proteasome for activated transcription of an ERE-driven luciferase reporter. **a** MS data depicted as a heatmap (displayed as in Fig. 1c) for ESR1-YAP1-dependent coactivators recruited to EREs from HNE. Recombinant purified WT or ESR1-YAP1 proteins were added to duplicate ERE DNA pulldown reactions. Fold change cutoff was ≥1.5 for enrichment over unliganded WT ERα. *ESR1 normalized ESR1 (see Supplementary Table 2). **YAP1 YAP1 corrected for ESR1. **b** Immunoblotting validation of the 26S proteasome being recruited to ESR1-YAP1. Independent ERE DNA pulldown samples were used to detect proteins recruited to ESR1-YAP1 compared to WT with/without E2 (i) or compared to WT or Y537S ERα (ii). Levels of ERα bound to the EREs were determined with an ERα antibody recognizing an N-terminal (N-term) epitope. TBP served as a loading control. 3% HNE, 3% of the starting HeLa S3 NE employed in the ERE DNA pulldown. **c** A 26S proteasome inhibitor, MG132, reduces ESR1-YAP1 transcriptional activity on an ERE-driven luciferase reporter. HeLa cells grown in phenol red-free, charcoal-stripped media were co-transfected with a vector expressing YFP-tagged ESR1-YAP1 protein and pERE-E1b-luc. Cells were then treated with vehicle (0.1% DMSO) or 1 μM MG132 for overnight. Luciferase activity (*RLU* relative light units) was assayed from whole-cell lysates. Data are represented as mean ± SEM (*n* = 3); ***p < 0.001

We next investigated co-regulator recruitment to ESR1-YAP1, which contains the N terminus and DBD, but lacks the LBD, of ERα fused in-frame to the C terminus of the Yes-associated protein 1^11^ (Fig. 1a). ESR1-YAP1 promotes high levels of expression of an ERE-dependent reporter without E2 (Fig. 1b and Supplementary Figure 6a). We compared co-regulator recruitment to either purified WT ERα or ESR1-YAP1 bound to ERE DNA in the absence of E2 (Fig. 6a). After normalization for ERα-binding differences, the ESR1-YAP1 protein did not recruit more SRC-3 and KMT2D as compared to WT ERα, unlike the LBD ERα mutants (validated in Fig. 1d). Instead, the ESR1-YAP1 protein recruited many subunits of the 26S proteasome (Fig. 6a). Immunoblotting validated enhanced proteasome recruitment (SUG1/PSMC5 and 20S C2/PSMA1) of ESR1-YAP1 vs. WT ERα, even with E2 (Fig. 6b). We further validated that the 26S proteasome was recruited to ESR1-YAP1 in E2-deprived T47D stable cell line [11] extracts (Supplementary Figure 8a).

The 26S proteasome plays a role in WT ERα degradation that is linked with the receptor’s ability to activate ERE-driven reporter genes [39–41]. We thus tested whether a proteasome inhibitor, MG132, would similarly inhibit ESR1-YAP1 transcriptional activity. Indeed, MG132 treatment of cells transfected with an ERE-driven reporter and an ESR1-YAP1 expression plasmid reduced Luc activity, as compared to the vehicle control (Fig. 6c). Interestingly, MG132 or the FDA-approved proteasome inhibitor, bortezomib, could inhibit the transcriptional activity of a GAL4 DBD-YAP1 fusion (Supplementary Figure 8b).

### Proteasome activity is important for ESR1-YAP1-mediated cell growth and gene expression

**Fig. 7:**
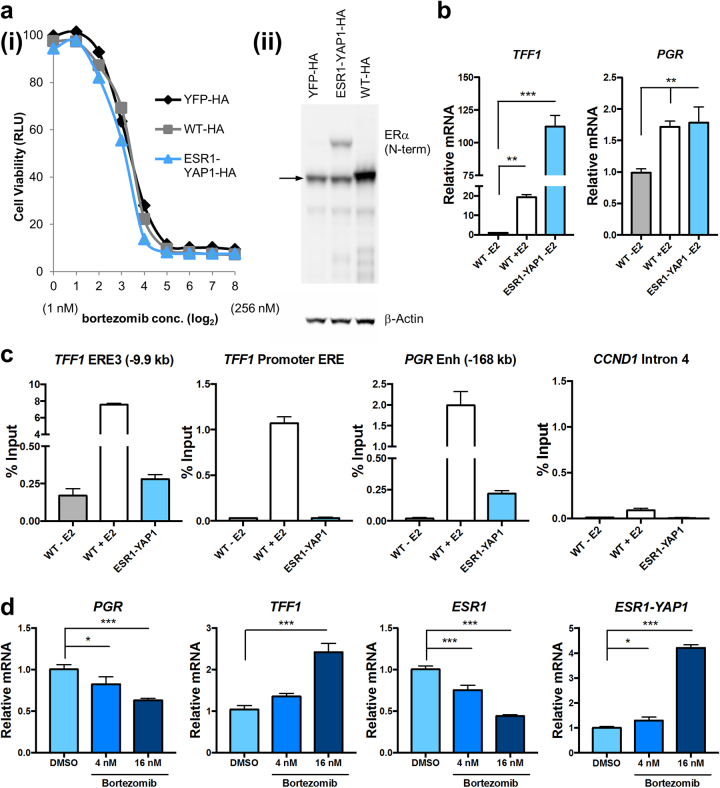
Proteasome inhibition reduces viability of breast cancer cells expressing the ESR1-YAP1 fusion and modulates ESR1-YAP1 target gene expression. T47D cell lines expressing these constructs were grown in phenol red-free, charcoal-stripped media for at least 2 days. **a** Increasing concentrations of bortezomib reduce cell viability of HA epitope-tagged YFP, WT ERα, and ESR1-YAP1 stably expressing T47D cell lines. (i) Cell viability was measured (via Cell Titer Glo as RLU) after 3 days of bortezomib or vehicle (DMSO) treatments. Data are represented as mean ± SEM (*n* = 3). (ii) Expression levels of ERα proteins were assayed in whole-cell extracts made from HA-tagged YFP, WT ERα, and ESR1-YAP1 expressing T47D cell lines. The N-terminal ERα antibody was used to detect endogenous ERα (denoted by →) as well as the ESR1-YAP1 fusion. β-actin served as a loading control. **b** Expression of HA-tagged ESR1-YAP1 activates expression of two classical ERE-containing target genes as compared to E2-deprived HA-tagged WT ERα. WT ERα-expressing cells were treated with/without 10 nM E2 for 24 h. Relative levels of *TFF1* or *PGR* mRNAs were determined by RT-qPCR using *ACTB* mRNA as the normalizer. Data are represented as mean ± SEM (*n* = 3); **p < 0.01; ***p < 0.001. **c** ESR1-YAP1 directly occupies certain EREs of the *TFF1* and *PGR* genes. Where indicated, HA-tagged WT ERα cells were treated with 100 nM E2 for 45 min before ChIP assays. ChIP-qPCR assays employed an antibody against HA to IP the HA-tagged ERα proteins and associated DNA. Representative data were plotted relative to percentage of starting input chromatin, which was represented as the mean of triplicate qPCR reactions ± SEM. *CCND1* gene intron 4 served as a negative control region. Supplementary Figure 8d shows a repeated ChIP assay. **d** Proteasome inhibitor treatment of T47D cells expressing HA-tagged ESR1-YAP1 modulates ERE-containing target gene expression. Cells were treated with vehicle (0.1% DMSO) or 4 or 16 nM bortezomib for 17 h. Relative levels of *PGR, TFF1*, *ESR1*, or *ESR1-YAP1* mRNAs were determined by RT-qPCR using *GAPDH* mRNA as the normalizer. Data are represented as mean ± SEM (*n* = 3); *p < 0.05; ***p < 0.001

We next tested the functional significance of the ESR1-YAP1: 26S proteasome interaction in T47D stable lines expressing HA-tagged YFP, WT ERα, or ESR1-YAP1 proteins (Fig. 7aii and Supplementary Figure 8c). Increasing concentrations of bortezomib treatment for 3 days efficiently reduced the growth of all three T47D lines compared to the vehicle control (Fig. 7a,i).

We next wanted to define the effect of ESR1-YAP1 expression in T47D cells grown in an E2-deprived state on ERα target gene expression. The ESR1-YAP1 fusion promoted the expression of two target genes, *TFF1* and *PGR*, significantly above the level of unliganded WT ERα-expressing cells (Fig. 7b), even though much less ESR1-YAP1 fusion was expressed (Fig. 7a, ii and Supplementary Figure 8c). The regulation of these two genes is likely direct, as ChIP assays revealed ESR1-YAP1 occupancy at two defined ERE enhancer-like sequences [35, 42] (Fig. 7c and Supplementary Figure 8d).

The effect of proteasome inhibition on *TFF1* and *PGR* gene expression in E2-deprived ESR1-YAP1-expressing cells was tested by treating cells with bortezomib (Fig. 7d). Proteasome inhibition had both a dose-dependent and gene-specific effect on the ESR1-YAP1 targets, decreasing *PGR* and increasing *TFF1*, which resembles the effect of proteasome inhibitors on these genes after E2 treatment of MCF-7 cells [43–46]. Bortezomib decreased endogenous *ESR1* mRNA expression, consistent with prior reports [43, 47]. Finally, we observed that bortezomib stimulated *ESR1-YAP1* mRNA expression (driven by the CMV promoter), which may explain why bortezomib did not severely reduce ESR1-YAP1 cell viability as compared to overexpressed WT ERα (Fig. 7a). Thus, the proteasome modulates ESR1-YAP1 target genes and cell growth, suggesting a new approach for treating tumors bearing this class of resistance mutation.

### Potential clinical relevance of mutant ERα-binding coactivators

To investigate whether the expression levels of the coactivators identified in this study correlate with patient outcomes, we queried two existing expression data sets—the Symmans Breast 2 ER-positive tamoxifen-treated patients [48] or ER-positive breast cancer patients treated with endocrine therapy (in KM plotter [49]; Supplementary Figure 9). In the Symmans data set, we found significantly higher KMT2D mRNA levels in patients who had a metastatic occurrence after 3 years of tamoxifen treatment vs. those that had not (panel a). We also found that higher SRC-3 and proteasomal subunit mRNA levels correlated with reduced survival from distant metastasis (panels b, c). In the KM plotter analysis, we found that higher KMT2D, but not KMT2C, mRNA significantly correlated with reduced recurrence-free survival (panel d). Finally, we assayed a metastatic breast cancer mutation/amplification database [50] and found that the ESR1 LBD mutations were not present in patients with mutations in either KMT2C or KMT2D genes (panel e).

## DISCUSSION

**Fig. 8:**
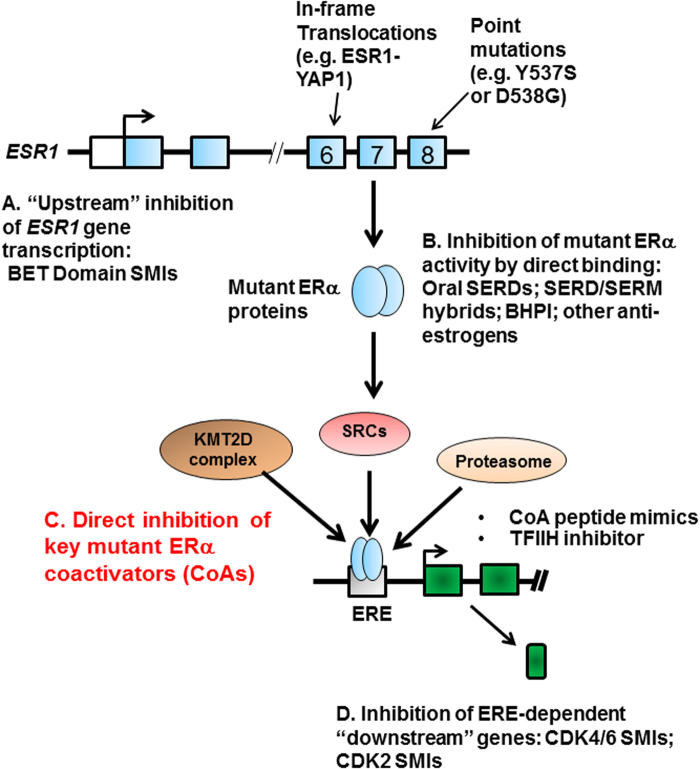
Potential ways to inhibit mutant ERα-driven breast cancer cell growth. In metastatic breast cancer, the *ESR1* gene encoding ERα is mutated in exon 6 (by translocations creating in-frame fusion proteins such as ESR1-YAP1) or in exon 8 (by point mutation in the LBD creating Y537S or D538G ERα mutants). While other laboratories have published pharmacological inhibitor strategies for reducing *ESR1* mutant gene transcription (a), for inhibiting the LBD mutant ERα proteins directly (b), and for inhibiting downstream gene products whose expression is driven by LBD mutant ERα proteins (d), we propose that inhibition of key mutant ERα coactivators, such as KMT2C/2D, SRC coactivators, and the proteasome, is a new therapeutic approach for inhibiting mutant ERα-driven breast cancer cell growth (c). See also Discussion

Different therapeutic approaches have been proposed to inhibit LBD mutant ERα proteins in breast cancers (Fig. 8) [23, 26, 27, 29–31, 51–54]. However, resistance to these therapies will occur. We envisioned that by defining the co-regulator “complexome” of each ESR1 mutant protein we could identify new potential therapeutic targets (Fig. 8c).

We identified coactivators that exhibit enhanced binding to ERα mutant proteins. Consistent with prior literature [7, 9, 14, 18, 19, 55, 56], SRCs display enhanced recruitment to EREs bound by the two LBD ERα mutants as compared with unliganded WT ERα. Since all three SRCs are recruited to the two LBD ERα mutants, therapeutic intervention likely must be directed toward all three proteins. Accordingly, we show that a pan-SRC SMI can effectively inhibit LBD ERα mutant activity and breast cancer cell growth (Fig. 2). Furthermore, combining pan-SRC SMI and oral SERD treatments have a synergistic effect on both LBD mutant transcriptional activity and cell growth (Fig. 2), and, more importantly, Y537S ERα-expressing PDX tumor growth (Fig. 3).

Our results further reveal coactivator complexes preferentially interacting with ERα mutants. First, we show that KMT2C/2D H3K4 methyltransferases are preferentially recruited to Y537S (Fig. 1c, Supplementary Figure 2a). These complexes were previously shown to coactivate E2-bound WT ERα in MCF-7 cells [57–60]. Although Y537S and D538G are one amino-acid residue apart in helix 12, it is striking that the KMT2C/2D complex is preferentially recruited to Y537S, and that it promotes the growth of Y537S ERα-expressing cells (Fig. 5e, Supplementary Figure 5). Detailed structural analysis is needed to examine how such coactivator-binding selectivity is achieved. As expression of Y537S ERα confers poor prognosis in metastatic breast cancer [16], our data suggest that developing targeted inhibitors of KMT2C/2D would be worthwhile for inhibition of Y537S ERα mutant activity. Furthermore, KMT2D is oncogenic in ER-positive breast cancers resistant to PI3Kα inhibitors [61].

Second, we have profiled coactivators bound to ESR1-YAP1, as it represents a “paradigmatic” gene fusion that activates ERE-driven transcription (Figs. 1b, 7b, Supplementary Figure 6). The ESR1-YAP1 fusion biochemically behaved in a distinct manner from the two LBD ERα mutants, as it displayed enhanced recruitment of the 26S proteasome (Fig. 6, Supplementary Figure 8a). Furthermore, proteasome activity is important for both ESR1-YAP1 transcriptional activity and growth of breast cancer cells (Figs. 6c, 7a, b). As the proteasome inhibitor bortezomib is in clinical trial for ERα-positive, metastatic breast cancer (NCT01142401), we propose that it may reduce ESR1-YAP1-mediated tumor growth. Recently, other *ESR1* gene fusions [20] have been identified in ER-positive metastatic disease and whether they also utilize the proteasome for their activity should be further investigated.

In summary, our study utilized an MS approach profiling three different mutant ERα proteins, which identified coactivators preferentially recruited to LBD mutants vs. an ESR1 gene fusion. We further showed that inhibition of these coactivators decreased ERE-driven transcription and reduced growth of breast cancer cells expressing these ERα mutants. Importantly, the combination of a pan-SRC inhibitor and oral SERD reduced the tumor growth of a human PDX expressing the Y537S ERα mutant, suggesting this as a potential new therapeutic strategy. We further identify additional potential therapeutic targets for Y537S ERα-expressing breast cancers (KMT2C/2D complexes) and for the ESR1-YAP1 fusion (26S proteasome) for which no prior targets were known. Together, our data support the idea that differential coactivator recruitment may be partly responsible for the ability of ERα mutant proteins to potentially drive metastatic breast cancer.

## Materials and methods

### Cell lines

Lines were obtained from BCM tissue culture core (originally from ATCC) unless otherwise indicated. STR profiling validated MCF-7 cell line authenticity. All lines tested negative for mycoplasma. MCF-7 and T47D cell lines stably expressing WT, Y537S, and D538G ERα were described [28]. C-terminal HA-tagged YFP, ESR1-WT, and ESR1-YAP1 lentiviral T47D cell lines were generated as in [11] using the pCD516B-2 vector (System Biosciences). Dox-inducible N-terminal FLAG-tagged WT, Y537S, and D538G ERα stable MCF-7 cell lines were constructed with pCW-FLAG-ERα lentiviruses. All lines were maintained in media containing 10% fetal bovine serum and switched to phenol red-free, hormone-depleted media before treatments.

### Plasmids

pCW-FLAG-WT, Y537S, or D538G were created from pCW-Cas9 [62] (Addgene). pFLAG-CMV constructs were made from pFLAG-CMV2 (Sigma). YFP-tagged ESR1-YAP1 was constructed by PCR of the ESR1-YAP1 open reading frame (ORF) [11], followed by ligation into digested pECFP-C1 (Clontech). pBIND-YAP1 (GAL4 DBD-YAP1 amino acid 230–504 fusion expression plasmid) was constructed by ligation into pBIND (Promega). All constructs were sequenced.

### ERE DNA pulldown assays

The ERE DNA pulldown was described [21]. One microgram of purified receptor was added to 2–2.5 mg HNE and 15 pmol 4xERE-E4 DNA immobilized on Dynabeads M-280 streptavidin (Invitrogen). For Supplementary Figure 8a, NE was made as described [63]. For direct interaction assays, 10 μl of the purified recombinant KMT2D “fusion” complex [22] was incubated with 0.25 μg of purified ERα for 1.5 h at 4 °C followed by washes and elution [21].

### Mass spectrometry

MS was performed and analyzed as described [21]. All data sets are summarized in Supplementary Tables 1, 2, and 7. The raw MS proteomics data were deposited to the ProteomeXchange Consortium (http://proteomecentral.proteomexchange.org), data set identifier: PXD005887.

### Recombinant ERα proteins

Flag-tagged ERα ORFs were expressed using the BaculoDirect N-terminal Expression kit (Invitrogen). Infected *Sf*9 cells were lysed in lysis buffer (50 mM Tris-Cl, pH 7.5, 500 mM NaCl, 15% glycerol, 0.01% NP40, 10 mM β-glycerophosphate and protease inhibitor cocktail (Roche)), followed by purification using anti-FLAG M2 antibody-conjugated beads and 3 × FLAG peptide (Sigma).

### Co-immunoprecipitations

Dox-treated MCF-7 cells expressing WT or LBD mutant ERα proteins were lysed in NETN buffer [21]. Three milligrams whole-cell extract was incubated for 4 h at 4 °C with 5 μg mouse anti-FLAG-pre-bound Protein G Dynabeads (Invitrogen) and washed with NETN and PBS.

### Reagents

Dimethyl sulphoxide, 17β-estradiol (E2), 4-hydroxytamoxifen, ICI182,780 (ICI), MG132, and Dox were purchased from Sigma. Bortezomib (Selleckchem), AZD9496 (MedChemExpress), and GDC0180 (Active Biochem) were from listed vendors. SI-1 and SI-2 have been described [25]. Antibodies are listed in Supplementary Table 3.

### Luc reporter assays and siRNA transfections

For ERE-Luc assays, HeLa cells were transfected with pERE-E1b-Luc [39] and ERα expression vector using Lipofectamine LTX (Invitrogen), and lysed in Glo Lysis Buffer (Promega). For GAL4-Luc assays, cells were transfected with pBIND-YAP1 and pG5luc (Promega). Luc activity was measured on a Berthold luminometer as described [64]. Cells were co-transfected with pERE-E1b-luc, ERα expression plasmids, and siRNAs using Trans-IT-TKO (Mirus Corp.) or RNAimax (Invitrogen). siRNAs are listed in Supplementary Table 3.

### Cell viability and soft agar assays

Cell viability or soft agar anchorage-independent cell growth was measured after 3 days by either a CellTiter-Glo® Luminescent assay (Promega) or by a MTT assay [65].

### Chromatin immunoprecipitation

Cells in hormone-depleted media were subject to ChIP assays performed using the EZ-ChIP kit (Millipore) as described [21]. See Supplementary Table 3.

### Calculations of synergism and IC_50_

Synergism between two drugs was defined as a combination index < 1 using CompuSyn (http://www.combosyn.com/) or Calcusyn (http://www.biosoft.com/w/calcusyn.htm) software [66]. IC_50_ values were determined by using Very Simple IC50 Tool Kit (http://www.ic50.tk/).

### Statistical analysis

*P* values were calculated using Student’s *t-*test (two-sample, two-tailed) to compare two means or ANOVA (ordinary, one-way) followed by adjusting for multiple comparisons using the Dunnett or Tukey method. *P* values < 0.05 were considered significant. For statistics, experiments were performed with three to eight biological replicates (see figure legends).

### Real-time reverse transcription

Total RNA was isolated with Direct-zol RNA MiniPrep (Zymo Research) or RNeasy (Qiagen) kits. cDNA synthesis and real-time reverse transcription (RT-qPCR) data analysis were carried out as described [64]. See Supplementary Table 3 for primers.

### PDX experiments

Experiments were carried out in accordance with protocol AN-1875 approved by the BCM Institutional Animal Care and Use Committee. WHIM 20 PDX was transplanted as described [11] into 4–5-week-old female SCID/Beige mice (Envigo). Mice were palpated semi-weekly, and tumor growth measured using calipers. When tumors reached ~350 mm^3^ (volume = *L*×((*W*×*W*)/2)), the mice were randomized into four treatment groups (*n* = 10 each): (1) AZD9496 5 days/week by oral gavage once daily, 5 mg/kg body weight (b.w.) [14, 26]; (2) SI-2 twice daily 5 days/week by intraperitoneal (i.p.) injection at 2 mg/kg b.w. [25]; (3) combination of both inhibitors as described; and (4) control PBS vehicle (oral and i.p.). Mice were sacrificed and tissues harvested at 4 months post transplantation. Tumor volume of eight mice per group (which completed at least 4.5 weeks of treatment) was analyzed. Representative tumors (*n* = 6/treatment) were lysed in RIPA buffer with phosphatase and protease inhibitors (Calbiochem). Hematoxylin and eosin staining and immunohistochemistry were performed by the Lester and Sue Smith Breast Center Pathology Core at BCM. After staining, images were captured on an OLYMPUS DP73 microscope. For BrdU analysis, 7 mg/ml BrdU (Sigma) was prepared in PBS and i.p. injected at 10 µl/g b.w. After 2 h, the mice were sacrificed (*n* = 8). Slides were stained by BD Pharmingen in Situ-Detection KIT II and were visualized with diaminobenzidine tetrahydrochloride (Dako), and counterstained with Harris hematoxylin. Images were analyzed in Matlab by the Integrated Microscopy Core at BCM. The investigators were not blinded to allocation during experiments and outcome assessment. No statistical methods were used to predetermine sample size estimate.

### Patient data analysis

Oncomine was used to query the Symmans Breast 2 data set. KM plotter (kmplot.com) was used to query the ER-positive patients treated with endocrine therapy. See Supplementary Figure 9 for more details.

## Electronic supplementary material

Supplementary Information clean

Supplementary Table 7 for Fig 1 and Supp Fig 1

Supplementary Table 7 for Supp Fig 2

Supplementary Table 7 for Fig 6
