# Supplementary material for: Proteomic profiling identifies key coactivators utilized by mutant ERα proteins as potential new therapeutic targets
Source: Oncogene. 2018 May 11;37(33):4581–98. doi: 10.1038/s41388-018-0284-2 (PMC6095836; doi:10.1038/s41388-018-0284-2)
Supplement: Supplementary file 1 — Supplementary Information clean [file 41388_2018_284_MOESM1_ESM.docx]

**Supplementary Information**

**Proteomic profiling identifies key coactivators utilized by mutant ERα proteins as potential new therapeutic targets**

Leah A. Gates, Guowei Gu, Yue Chen, Aarti D. Rohira, Jonathan T. Lei, Ross A. Hamilton, Yang Yu, David M. Lonard, Jin Wang, Shu-Ping Wang, David G. Edwards, Philip F. Lavere, Jiangyong Shao, Ping Yi, Antrix Jain, Sung Yun Jung, **Anna Malovannaya**, Shunqiang Li, Jieya Shao, Robert G. Roeder, Matthew J. Ellis, Jun Qin, Suzanne A. W. Fuqua, Bert W. O’Malley, and Charles E. Foulds

**List of Supplementary Material**

**1) Supplementary Figures**

- Supplementary Figure 1, related to Figure 1
- Supplementary Figure 2, related to Figure 1
- Supplementary Figure 3, related to Figure 2
- Supplementary Figure 4, related to Figure 3
- Supplementary Figure 5, related to Figure 4
- Supplementary Figure 6, related to Figure 5
- Supplementary Figure 7, related to Figure 5
- Supplementary Figure 8, related to Figures 6 and 7
- Supplementary Figure 9, related to Figure 8

**2) Supplementary Figure Legends**

- Supplementary Figures 1-9

**3) Supplementary Tables**

- Supplementary Table 1: Details on all mass spectrometry (MS) experiments, related to Figures 1c and 6a, and Supplementary Figures 1a, 1c, and 2a
- Supplementary Table 2: Details on how six N-terminal ESR1 peptides were used for normalization of MS data
- Supplementary Table 3: Details on antibodies used for immunoblotting and ChIP, primers and probes used for RT-qPCR, primers used for ChIP, and siRNAs
- Supplementary Table 4: Testing drug synergy between AZD9496 and SI-1 on inhibiting Y537S and D538 ERα-mediated transcriptional activity, related to Figures 2d and 2e
- Supplementary Table 5: Testing drug synergy between AZD9496 and SI-1 on reducing cell viability of WT and Y537S ERα expressing MCF-7 cells, related to Figure 2f
- Supplementary Table 6: Testing drug synergy between ICI 182,780 and SI-1 on reducing cell viability of WT and Y537S ERα expressing MCF-7 cells, related to Supplementary Figure 3e
- Supplementary Table 7: Excel file of all binding proteins in ERE DNA pulldown assays

**4) Supplemental References**

**1) Supplemental Figures**

**
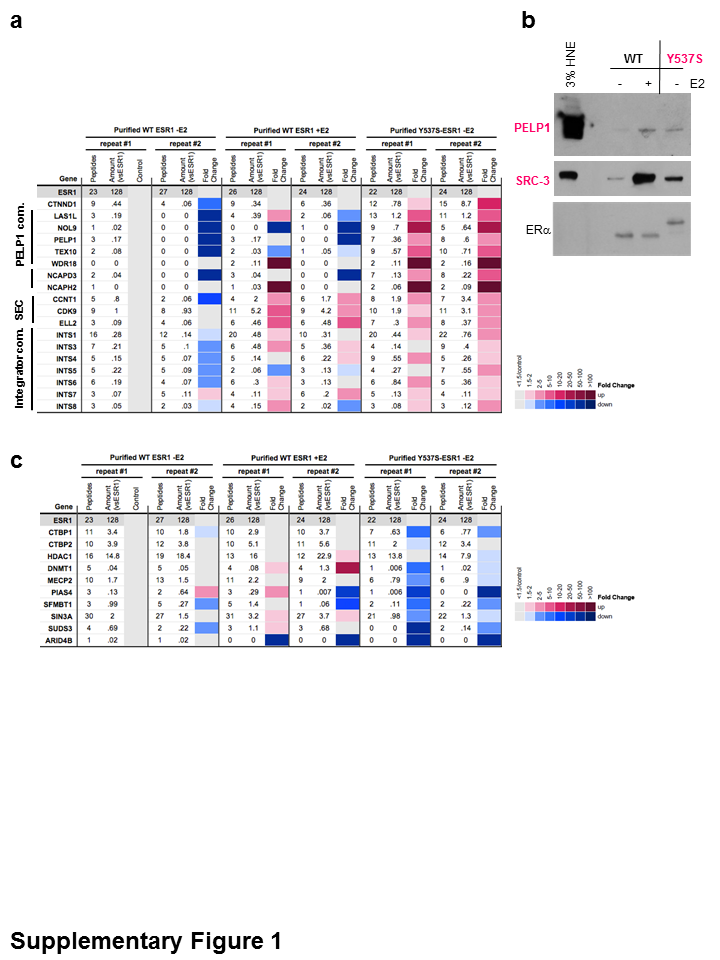
**

**
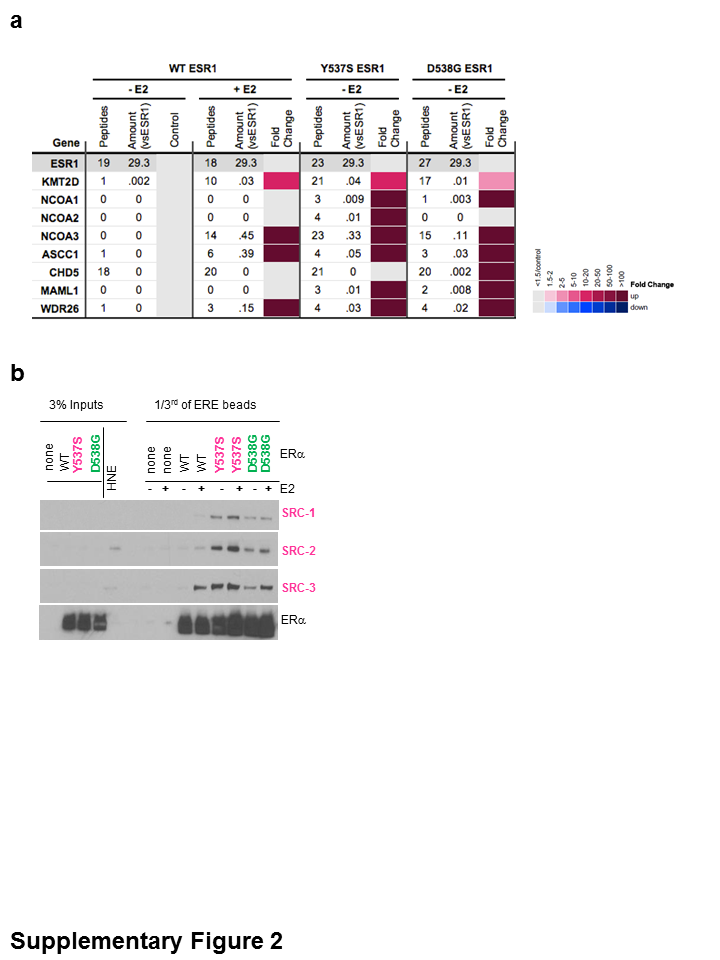
**

**
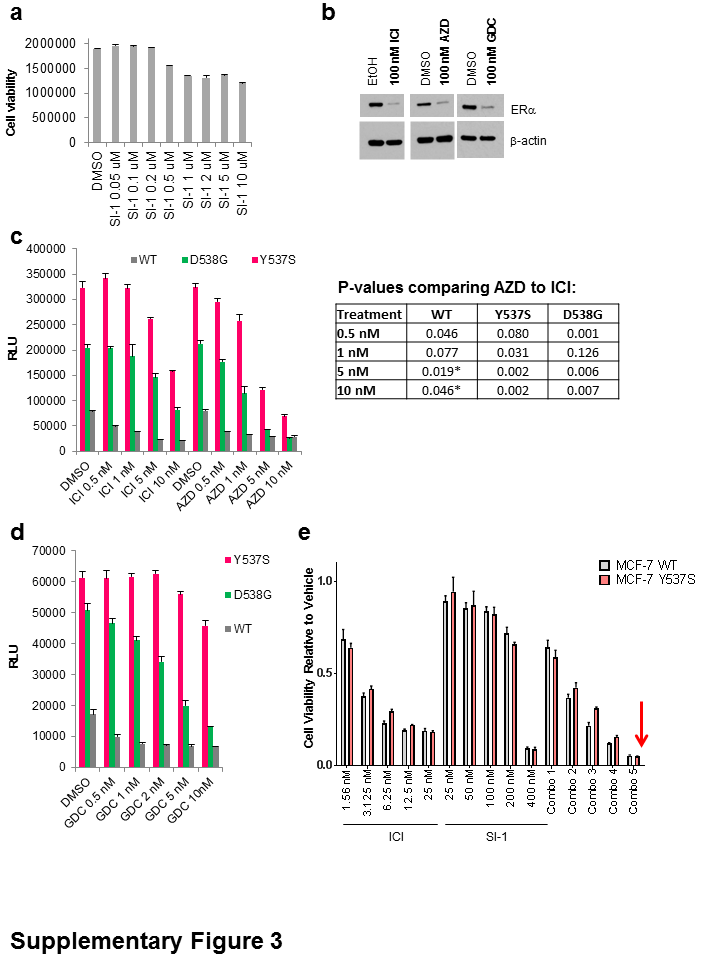
**

**
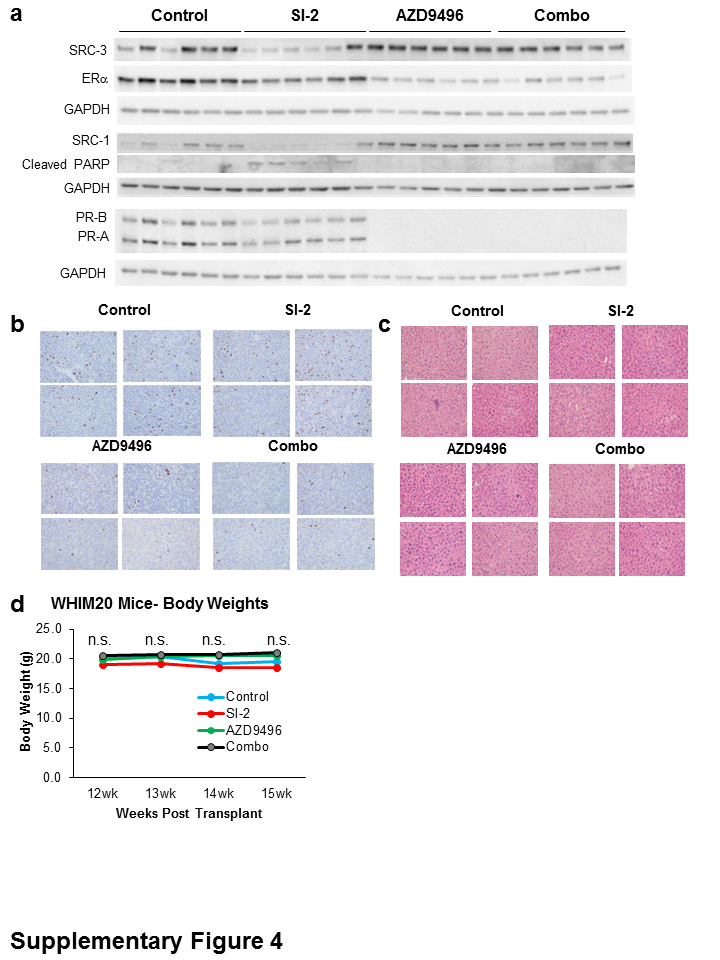
**

**
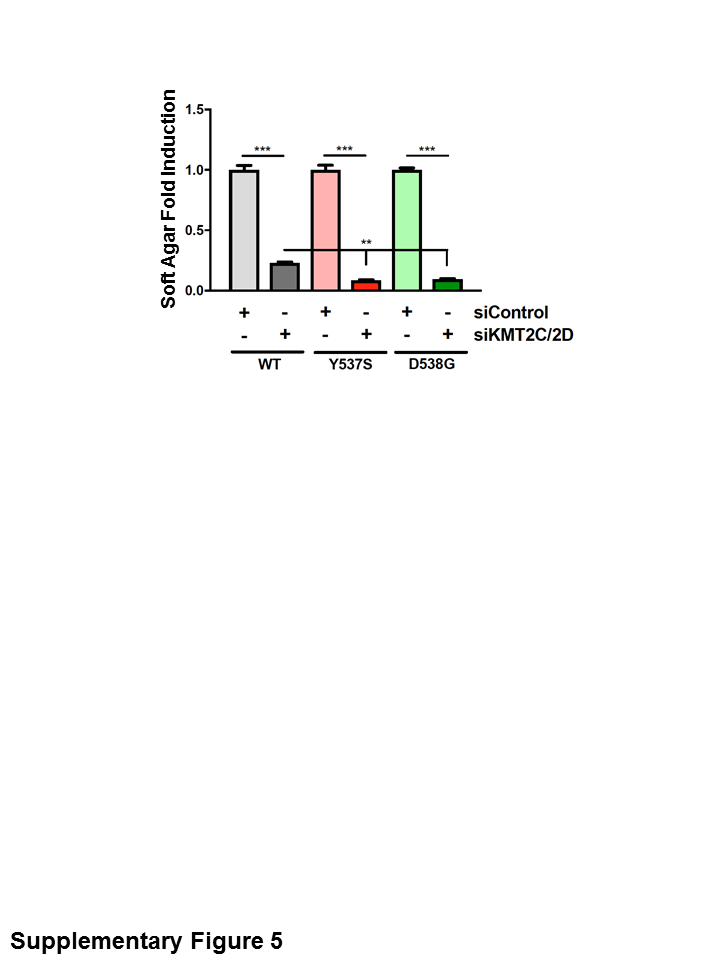
**

**
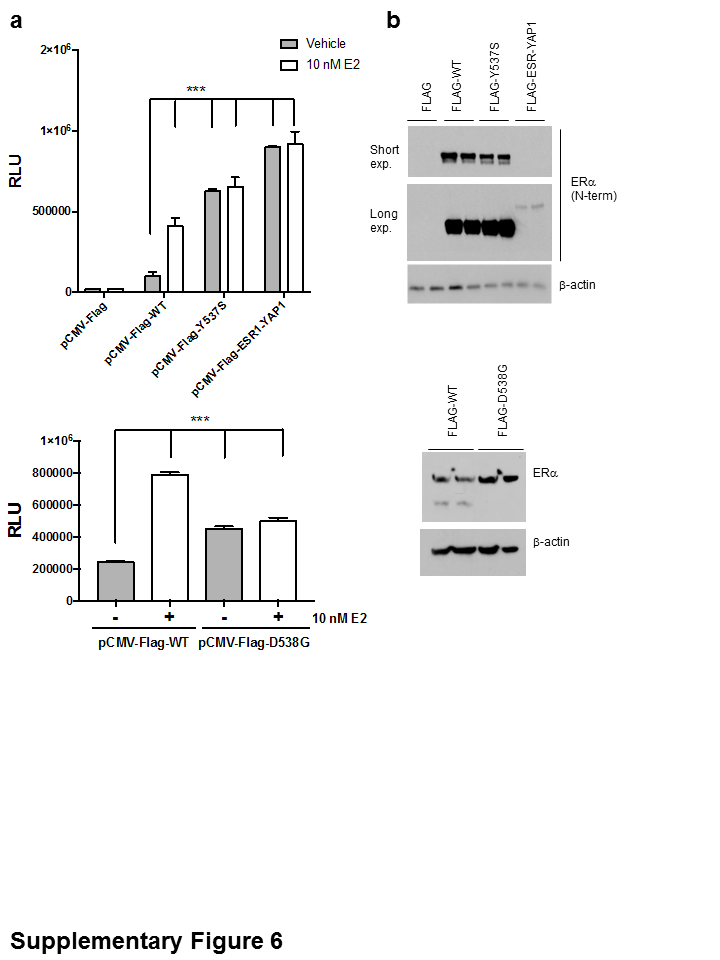
**

**
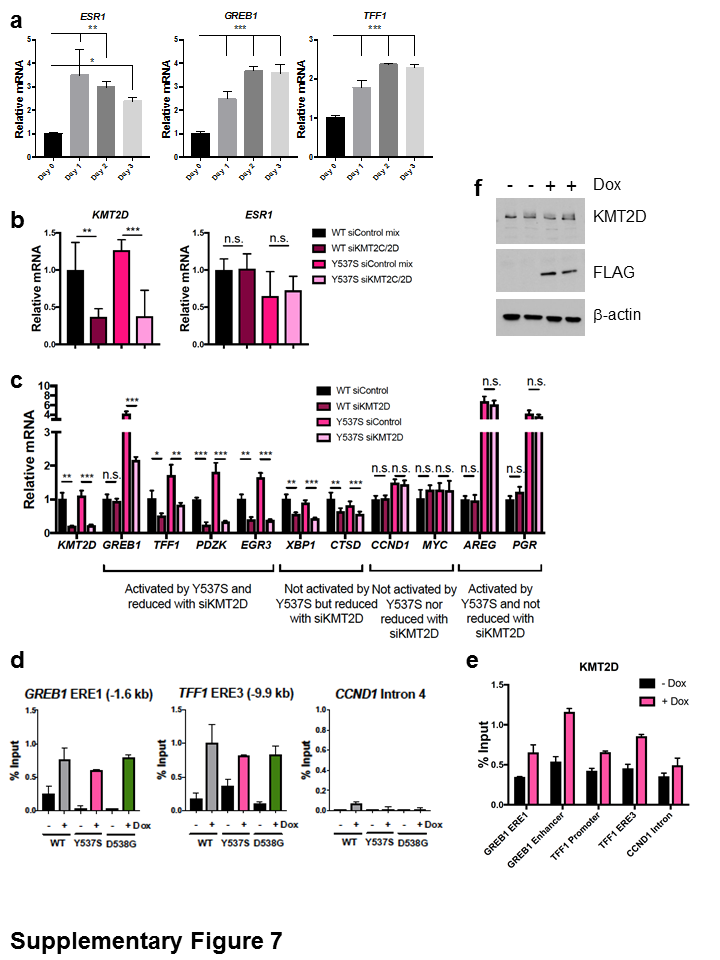
**

**
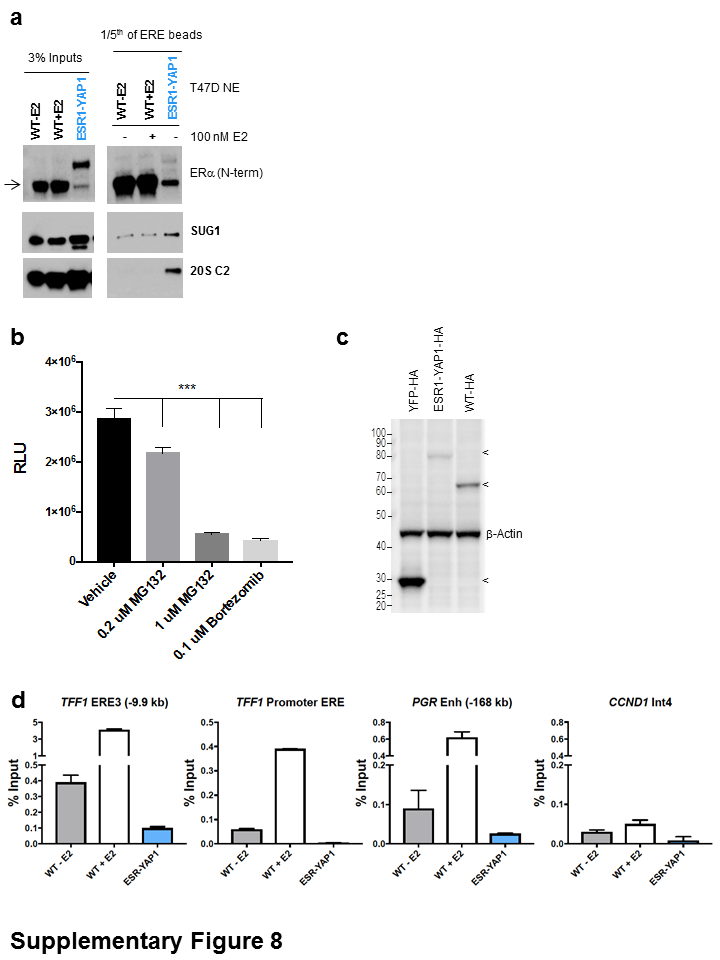
**

**
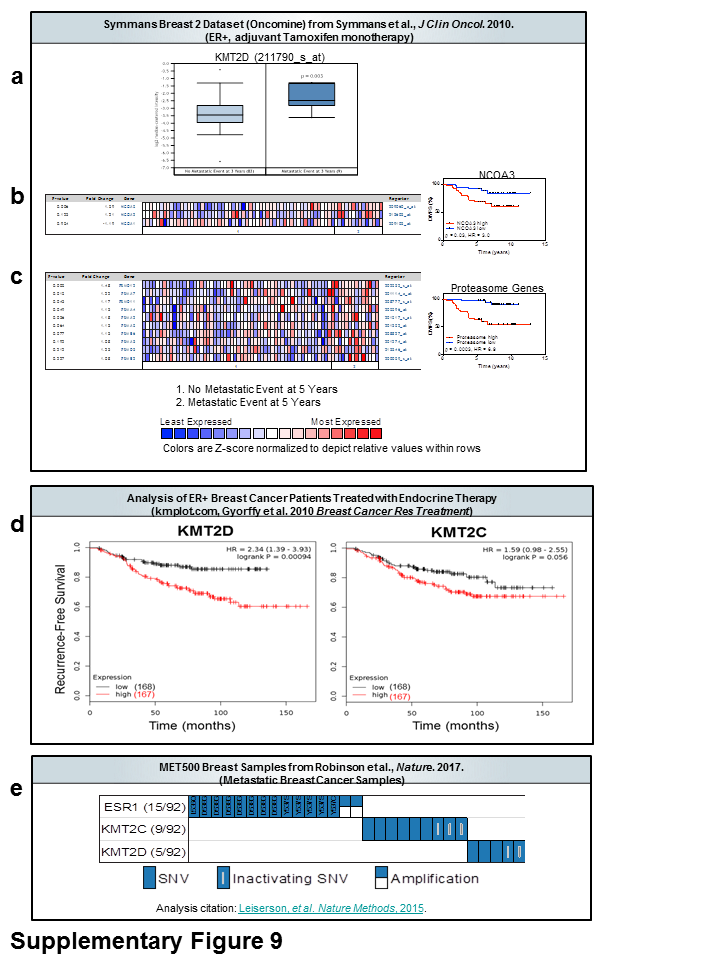
**

**2) Supplementary Figure Legends**

**Supplementary Figure 1.** Mass spectrometric (MS) identification of additional co-regulators bound with WT or Y537S ERα proteins on EREs. We interrogated the same MS dataset as in Figure 1c for additional co-regulators, and compared the recruitment to Y537S ERα with unliganded WT ERα for ESR1 binding. (a) MS heatmap of additional classes of coactivators detected by DNA pulldowns. Com = complex. NCAPD3 and NCAPH2 are subunits of the condensin complex. (b) Immunoblotting validation of PELP1 as having ligand-independent recruitment to Y537S ERα. (c) Analysis of major classes of corepressors (see[^1^](#_ENREF_1) for details) detected by DNA pulldowns-MS to have reduced recruitment to the Y537S ERα. The data is shown as a heatmap with the darker blue indicating reduced binding.

**Supplementary Figure 2.** Comparison of co-regulators recruited with WT, Y537S, and D538G ERα in DNA pulldown assays. The three ERα proteins were expressed in 293T cells, and whole-cell extracts were incubated with EREs before HNE addition. After beads were washed, proteins were identified by MS and normalized to amount of ESR1 bound. (a) MS heatmap of co-regulator proteins detected by DNA pulldown as compared to unliganded WT ERα. (b) All three SRC proteins are predominantly enriched with Y537S ERα protein, then with D538G ERα, as compared to unliganded WT ERα protein by immunoblotting.

**Supplementary Figure 3.** Control experiments on effects of a “pan-SRC” inhibitor SI-1 and SERDs on HeLa cells and MCF-7 cells expressing WT or point mutant ERα. (a) Cell viability (measured by Cell Titer Glo as RLU) of HeLa cells after treatment with increasing concentrations of the SRC inhibitor SI-1 (0.05 - 10 μM) after 16 hr exposure was only marginally reduced (1.2 fold with 0.5 μM SI-1; 1.6 fold with 10 μM SI-1). DMSO served as the vehicle control. Data are represented as mean + SEM (n=3). (b) Two oral SERDs reduce endogenous ERα protein levels in MCF-7 cells similarly to ICI 182,780 (ICI) treatment. Cells were treated with vehicle (ethanol, EtOH or DMSO) or 100 nM ICI, 100 nM AZD9496 (AZD), or 100 nM GDC-0810 (GDC) for 4 hours. Cells were then harvested, whole-cell extracts made in NETN, and immunoblotting for ERα (Santa Cruz HC-20 antibody) or β-actin (loading control) was performed. (c) AZD, like ICI, effectively reduces WT, Y537S and D538G ERα transcriptional activities in HeLa cells. Cells were transfected with YFP-tagged ERα expression vectors and pERE-E1b-luc in phenol red-free, charcoal stripped media. After overnight treatment with the drugs, luciferase activities (RLU) were measured in cell lysates as in Figure 2c. Data are represented as mean + SEM (n=3). To assay the statistical significance of AZD versus ICI in reducing ERα transcriptional activity, fold change of RLU was calculated for each concentration of AZD or ICI compared to their respective DMSO control values and P-values were calculated using the Student’s t test (right panel, table). WT ERα activity is sensitive to both ICI and AZD, and at 5 and 10 nM concentrations (indicated with *), ICI was more potent than AZD. (d) GDC reduces WT and D538G ERα transcriptional activities in HeLa cells, while having a marginal effect on Y537S ERα activity. Cells were transfected and data analyzed as above. Data are represented as mean + SEM (n=3). (e) ICI is more effective than the SRC SMI SI-1 at reducing cell viability of MCF-7 lentiviral transduced stably expressing WT and Y537S ERα lines. Cell viability was assayed by MTT assay after six days of treatment and data (n=3) was plotted relative to vehicle (DMSO) control. Synergism of ICI and SI-1 in reducing viability of Y537S ERα expressing cells was observed with combination treatment 5 (Combo 5: 25/400 nM ICI/SI-1) (shown as a red arrow) based on Calcusyn software calculations shown in Supplementary Table 6.

**Supplementary Figure 4.** Analysis of Y537S expressing PDX tumors subject to different treatment regimens. (a) Immunoblotting of ERα, SRC-1 and -3, and select markers of ERα activity (PR expression) and apoptosis (cleaved PARP). GAPDH serves as normalization control after Image J analysis (n=6 per group). (b-c) Histological staining of four representative tumor slides. (b) BrdU staining shows reduced proliferation in the AZD9496 and combination therapy groups. (c) H&E staining does not reveal any overt toxicity in host mouse livers between treatment groups. (d) The body weights of WHIM20 PDX mice (n=10 at weeks 12 and 13; n=5-10 at later weeks) do not significantly change with inhibitor treatments. n.s. = not significant.

**Supplementary Figure 5.** Knockdown of KMT2C and KMT2D reduces WT, Y537S, and D538G ERα - mediated growth on soft agar in lentiviral transduced stably overexpressing MCF-7 cells. Non-targeting (siControl) or KMT2C/2D targeting siRNAs were transfected into WT, Y537S, or D538G ERα MCF-7 cells at a final concentration of 100 nM (50 nM each) in complete growth media in six well plates. One day later, cells were re-plated in 24 well plates, and after seven days, colonies formed in soft agar were visually (or automatically) counted (using GelCount) and quantified as in Figure 4c. For each cell line, the siControl value was set to 1. Data are represented as mean + SEM (n=4). **, p-value<0.01 and ***, p-value<0.001 by one-way ANOVA comparing every mean to each other.

**Supplementary Figure 6.** A N-terminal FLAG epitope tag does not affect WT, Y537S, or D538G ERα or ESR1-YAP1 transcriptional activity. (a) HeLa cells grown in phenol red-free, charcoal-stripped media were co-transfected with pERE-E1b-luc and a FLAG-tagged ERα expression plasmid (pCMV-FLAG). WT ERα expressing cells were further treated overnight (-/+) 10 nM E2. Cell lysates were assayed for luciferase activities (RLU). Data are represented as mean + SEM (n=3). ***, p-value <0.001 by one-way ANOVA comparing other means to FLAG-tagged WT without E2 treatment. (b) Immunoblotting to determine FLAG-tagged ERα protein expression levels (WT, Y537S, D538, and ESR1-YAP1) in transfected HeLa cells. In parallel to above luciferase assays, cells were transfected (in duplicate), but not treated with E2. Whole-cell extracts were prepared in NETN, followed by running on SDS-PAGE and immunoblotting with ERα N-terminal antibody (top panel) or ERα HC-20 antibody (bottom panel) with β-actin serving as a loading control. Exp., exposure of film after ECL development.

**Supplementary Figure 7.** Control experiments for Dox-inducible WT and LBD mutant ERα MCF-7 cell lines. (a) As little as two days treatment with Dox in the inducible FLAG-tagged Y537S ERα MCF-7 cell line was sufficient to maximally stimulate expression of two classical ERα target genes. After three days in charcoal-stripped media, cells were treated for the indicated times with 0.5 μg/ml Dox (d1, one day, d2; two days, d3; three days) or without (d0). RNA was isolated from cells and analyzed by RT-qPCR for the relative mRNA levels of *ESR1* (to confirm induction of Y537S ERα), *GREB1*, and *TFF1*. Relative levels of these mRNAs were determined using *ACTB* mRNA as the normalizer. Data are represented as mean + SEM (n=3). *, ** and ***, p-values <0.05, <0.01, and <0.001 by one-way ANOVA, as compared to untreated d0 control. (b-c) Analysis of gene expression after siRNA knockdown. Cell lines were grown in phenol red-free, charcoal-stripped media and transfected with siRNAs. On the next day, 0.5 μg/ml Dox was added for two more days. RNA was then isolated, and RT-qPCR for the relative mRNA levels was determined using *ACTB* mRNA as the normalizer. Data are represented as mean + SEM (n=3). *, ** and ***, p-values <0.05, <0.01, and <0.001, respectively, and n.s. = not significant (p>0.05), by Student’s t-test, as compared to non-targeting siControl or siControl mix. (b) Transfection of WT and Y537S ERα Dox-inducible MCF-7 cell lines with siRNAs targeting KMT2C and KMT2D did not affect *ESR1* mRNA levels. (c) Knockdown of KMT2D alone significantly reduces select gene expression in Dox-treated WT or Y537S ERα cell lines. (d) A repeated ChIP-qPCR experiment validates Figure 5g data that Dox-induced FLAG-tagged WT, Y537S, and D538G ERα proteins occupy EREs of *GREB1* and *TFF1* genes, but not a region of intron 4 of the *CCND1* gene in MCF-7 cells. ChIP assays employed an antibody against FLAG to IP the FLAG-tagged ERα proteins and associated DNA. Representative data was plotted relative to percentage of starting input chromatin and is represented as mean of triplicate qPCR reactions + SEM. (e) A repeated ChIP-qPCR experiment validates Figure 5h data that KMT2D occupies EREs of *GREB1* and *TFF1* genes in Dox-dependent manner correlating with increased Y537S ERα occupancy. Representative data was plotted as above and *CCND1* gene intron 4 served as a negative control region. (f) Expression of Dox-induced FLAG-tagged Y537S ERα in MCF-7 cells did not affect the endogenous KMT2D protein level. Y537S ERα expression was induced by 0.5 μg/ml Dox addition to cells grown in phenol red-free, charcoal-stripped serum containing media. After three days minus or plus Dox, duplicate cells in 10 cm dishes were harvested in NETN lysis buffer. Protein lysate was electrophoresed on SDS-PAGE and immunoblotted with antibodies to KMT2D (Millipore), FLAG, and β-actin (as a loading control).

**Supplementary Figure 8.** Control experiments showing proteasome subunit enrichment with ESR1-YAP1 from T47D cells, GAL4-YAP1 transcriptional activity inhibition by proteasome inhibitors, and immunoblotting of HA-tagged YFP, WT ERα, or ESR1-YAP1 stably expressing T47D cells. (a) An independent experiment employing NEs of lentiviral transduced T47D cells expressing FLAG-tagged WT or ESR1-YAP1 fusion proteins (previously described in[^2^](#_ENREF_2)) confirms proteasome enrichment with ESR1-YAP1 bound to EREs. Cell lines were grown in phenol red-free, charcoal stripped RPMI media for 7 days. Only the ESR1-YAP1 T47D cells were treated with 100 nM ICI for 3 hours to reduce the level of endogenous ERα prior to ERE DNA pulldowns (see 3% inputs). NEs from either WT ERα or ESR1-YAP1 expressing cells (1 mg each) were added to biotinylated 4xERE beads, washed, and bound proteins were eluted in SDS-sample buffer and processed for MS. 3% inputs represents 3% of NE added to beads (left panel). T47D WT ERα cells were treated with/without 100 nM E2 for 1 hour prior to small-scale nuclear extraction. Antibodies used: ERα N-terminal antibody, SUG1 (gene symbol: PSMC5) and 20S C2 (gene symbol: PSMA1). (b) 26S proteasome inhibitors, MG132 and bortezomib, reduce transcriptional activity of a GAL4 DBD- C-terminal YAP1 fusion protein on a GAL4-dependent luciferase reporter (pG5luc). HeLa cells grown in charcoal-stripped media were transfected with a vector expressing GAL4 DBD – C-terminal residues of YAP1 found in the ESR1-YAP1 fusion (230-504 amino acids[^2^](#_ENREF_2)). Cells were treated for 18 hours with 0.2 or 1 μM MG132, 0.1 μM bortezomib, or 0.1% DMSO (vehicle control). Luciferase activity (RLU) was assayed from whole-cell extracts. Data are represented as mean + SEM (n=3). *** = p-values <0.001 by one-way ANOVA, as compared to the vehicle control. (c) Immunoblotting with an anti-HA epitope antibody confirms that ESR1-YAP1-HA was expressed at a lower level in lentiviral stably transduced T47D cells than WT ERα-HA cells. Expression levels of ERα proteins were assayed in whole-cell extracts and β-actin serves as a loading control. Arrowheads show the position and level of HA-tagged YFP, ESR1-YAP1, or WT ERα. Numbers on left-hand side of blot indicate protein size standards (kDa). (d) A repeated ChIP-qPCR experiment validates Figure 7c data that ESR1-YAP1 directly occupies enhancer EREs of the *TFF1* and *PGR* genes. ChIP-qPCR assays employed an antibody against HA to IP the HA-tagged ERα proteins and associated DNA. Representative data was plotted relative to percentage of starting input chromatin, which was represented as mean of triplicate qPCR reactions + SEM. *CCND1* gene intron 4 served as a negative control region.

**Supplementary Figure 9.** Expression of KMT2D, SRC-3 (NCOA3), and proteasomal genes are prognostic factors in endocrine therapy-treated ER+ human tumors and KMT2C and KMT2D aberrations are mutually exclusive from each other and ESR1 mutations. (a) Tamoxifen-treated patients with a metastatic event at 3 years after diagnosis had significantly higher levels of KMT2D transcripts compared to patients without a metastatic event at 3 years. Box represents interquartile range (IQR), line within the box represents the median, whiskers represent 1.5 X IQR, and dots represent outliers. P-value determined by Mann-Whitney test. (b) (Left) Transcript expression levels of transcriptional co-regulators in patients with and without a metastatic event at 5 years. P-value indicates significant fold change enrichment of NCOA3 expression in patients with a metastatic event at 5 years. (Right) Kaplan-Meier (KM) plots of Distant Metastasis-Free Survival (DMFS) in patients categorized as having high (red line) and low levels (blue line) of NCOA3 transcript levels as determined by above and below median NCOA3 gene expression, respectively. Log_2_-median centered gene expression values were used to generate figures. Gene expression values were used to generate the Kaplan Meier plot and defined as high = patients with above median gene expression, low = patients with below median gene expression. (c) (Left) Transcript expression levels of proteasomal subunits in patients with and without a metastatic event at 5 years. P-values indicate significant fold enrichment of PSMD12 and PSMA7 in patients with a metastatic event at 5 years. (Right) KM plots of DMFS in patients categorized as having high (red line) and low (blue line) proteasomal gene signature scores as determined by above and below median proteasomal signature scores, respectively. A proteasomal gene signature score for each patient was calculated by averaging gene expression for all proteasomal genes. Color scales for b-c are Z-score normalized to depict relative values within rows and cannot be used to compare values between rows (Oncomine). For all KM plots, black dots indicate censored patients and Hazard Ratio (HR) and associated P-values were determined by log-rank tests. (d) KM plotter was used to examine Recurrence-Free Survival (RFS) in patients with ER+ tumors treated with endocrine therapy expressing high and low levels of KMT2D or KMT2C transcript levels as determined by above or below median expression, respectively, for the gene being queried. High levels of KMT2D significantly associated with poorer RFS and there is a trend towards poorer RFS in patients whose tumors expressed high levels of KMT2C. Vertical lines indicate censored patients and hazard ratio with associated P-values determined by log-rank tests (KM plotter). (e) Incidence of ESR1, KMT2C, and KMT2D aberrations were examined in metastatic breast tumors. ESR1 point mutations clustering in the ligand-binding domain were the most prevalent and mutually exclusive from KMT2C and KMT2D aberrations. SNV = Single Nucleotide Variant. Inactivating SNV are predicted loss-of-function SNVs.

**3) Supplementary Tables**

**Supplementary Table 1:** Details on all mass spectrometry experiments.

| **EXP no** | **Cell** | **Genotype** | **Fraction** | **Treat-ment** | **293T Extract**  **Amount** | **Nuclear Extract Amount** | **Affinity**  **Name** | **Affinity**  **Amount** | **MS**  **Instrument** | **Bands** |
| --- | --- | --- | --- | --- | --- | --- | --- | --- | --- | --- |
| 9919 | HeLa S3 | WT (+ESR1 293T cell extract) | Nuclear Extract | none | 1 mg | 2 mg | 4xERE | 15 pmol | BCM-Elite-1 | 6 |
| 9920 | HeLa S3 | WT (+ESR1 293T cell extract) | Nuclear Extract | E2 (1 μM), water-soluble | 1 mg | 2 mg | 4xERE | 15 pmol | BCM-Elite-1 | 6 |
| 9921 | HeLa S3 | WT (+Y537S ESR1 293T cell extract) | Nuclear Extract | none | 1 mg | 2 mg | 4xERE | 15 pmol | BCM-Elite-1 | 6 |
| 9922 | HeLa S3 | WT (+D538G ESR1 293T cell extract) | Nuclear Extract | none | 1 mg | 2 mg | 4xERE | 15 pmol | BCM-Elite-1 | 6 |
| 11847 | HeLa S3 | WT (+purified ESR1) | Nuclear Extract | Ethanol as vehicle | N/A | 2.5 mg | 4xERE | 15 pmol | BCM-Elite-1 | 6 |
| 11848 | HeLa S3 | WT  (+purified ESR1) | Nuclear Extract | Ethanol as vehicle | N/A | 2.5 mg | 4xERE | 15 pmol | BCM-Elite-1 | 6 |
| 11849 | HeLa S3 | WT  (+purified ESR1) | Nuclear Extract | E2 (100 nM) in Ethanol | N/A | 2.5 mg | 4xERE | 15 pmol | BCM-Elite-1 | 6 |
| 11850 | HeLa S3 | WT  (+purified ESR1) | Nuclear Extract | E2 (100 nM) in Ethanol | N/A | 2.5 mg | 4xERE | 15 pmol | BCM-Elite-1 | 6 |
| 11853 | HeLa S3 | WT (+purified Y537S ESR1) | Nuclear Extract | Ethanol as vehicle | N/A | 2.5 mg | 4xERE | 15 pmol | BCM-Elite-1 | 6 |
| 11854 | HeLa S3 | WT (+purified Y537S ESR1) | Nuclear Extract | Ethanol as vehicle | N/A | 2.5 mg | 4xERE | 15 pmol | BCM-Elite-1 | 6 |
| 12236 | HeLa S3 | WT  (+purified ESR1) | Nuclear Extract | none | N/A | 2.5 mg | 4xERE | 15 pmol | BCM-Elite-1 | 6 |
| 12237 | HeLa S3 | WT (+purified ESR1) | Nuclear Extract | none | N/A | 2.5 mg | 4xERE | 15 pmol | BCM-Elite-1 | 6 |
| 12238 | HeLa S3 | WT (+purfiedESR1-YAP1) | Nuclear Extract | none | N/A | 2.5 mg | 4xERE | 15 pmol | BCM-Elite-1 | 6 |
| 12239 | HeLa S3 | WT (+purified ESR1-YAP1) | Nuclear Extract | none | N/A | 2.5 mg | 4xERE | 15 pmol | BCM-Elite-1 | 6 |

**Supplementary Table 2:** Area under the curve (AUC) values of six consistently observed N-terminal ESR1 peptides (bolded) were summated and then used to normalize bound proteins in ERE DNA pulldowns (as “vsESR1” scale) and for the corrected amounts of ESR1. This correction is most crucial for experiments #12238 and #12239, where straightforward implementation of the intensity-based absolute quantification (iBAQ) method underestimates quantity of ESR1 truncated by the YAP1 fusion. Three consistently observed C-terminal LBD ESR1 peptides that are lacking in the ESR1-YAP1 fusion are shown in grey color. To have comparable scales to original iBAQ-based estimates of protein amounts in each data set, “Scaled ESR1 AUC” and total protein quantities (“Scaled “vsESR1” Total Quantity”) were scaled toward the numerical range of the original iBAQ-based estimates.


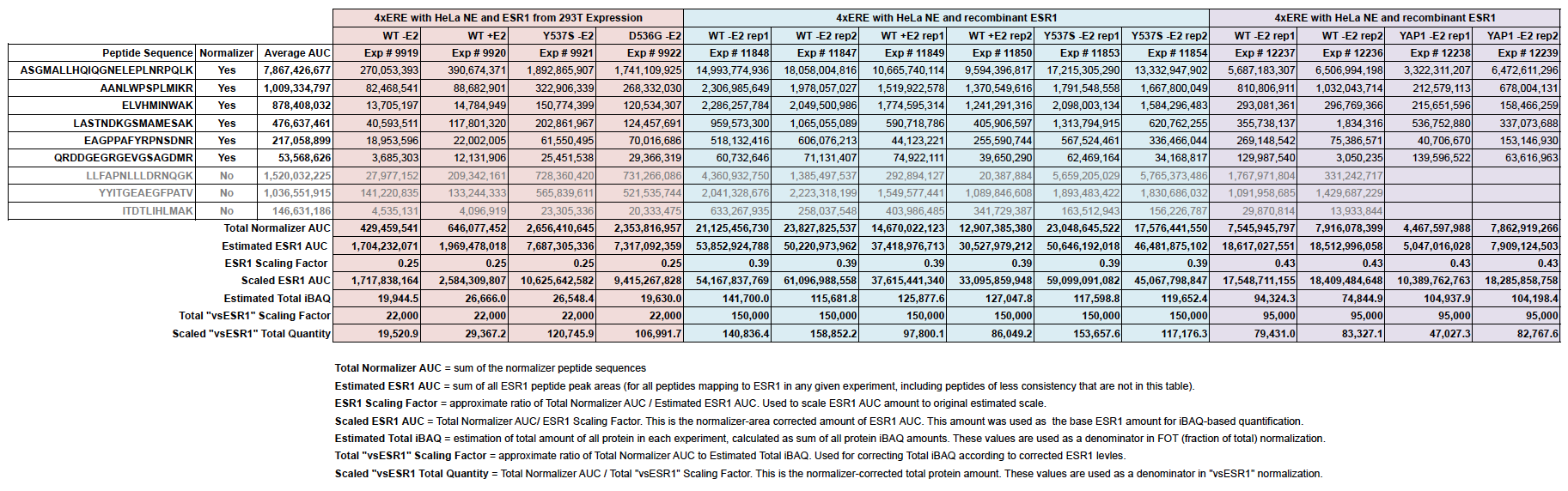


**Supplementary Table 3:** Details on antibodies used for immunoblotting and ChIP, primers and probes used for RT-qPCR, primers used for ChIP, and siRNAs.

**Antibodies used for Immunoblotting**

| **Antigen**  Official gene symbol (common name) | **Catalogue Number** | **Supplier** |
| --- | --- | --- |
| TBP (TBP) | sc-273 | Santa Cruz Biotechnology |
| ESR1 (ERα)  C-terminal | sc-543 | Santa Cruz Biotechnology |
| ESR1 (ERα)  N-terminal | 04-820 (Clone 60C) | Millipore |
| NCOA1 (SRC-1) | sc-32789 | Santa Cruz Biotechnology |
| NCOA2 (SRC-2) | 610985 | BD Biosciences |
| NCOA3 (SRC-3) | 611104 | BD Biosciences |
| NCOA3 (SRC-3) | Custom-made | BCM Monoclonal Antibody Core |
| ACTB (β-actin) | A5441 (Clone AC-15) | Sigma |
| ACTB (β-actin) | A5316 (Clone AC-74) | Sigma |
| (FLAG epitope) | F1804 | Sigma |
| (HA epitope) | 901513 (Clone 16B12) | BioLegend |
| PSMA1 (Proteasome Subunit 20S C2) | PA1-963 | Thermo Fisher Scientific |
| PSMC5 (SUG1) | A300-791A | Bethyl Laboratories |
| KMT2D (MLL4) | Custom-made | Dr. Kai Ge at NIH[^3^](#_ENREF_3) |
| KMT2D (MLL4) | ABE1867 | Millipore |
| PAGR1 (PA1) | A301-978A | Bethyl Laboratories |
| ASH2L | A300-489A | Bethyl Laboratories |
| GAPDH | sc-25778 | Santa Cruz Biotechnology |
| Cleaved PARP | 5625S | Cell Signaling Technology |
| PELP1 | A300-180A | Bethyl Laboratories |
| PGR (PR) | 1294 | Monoclonal ab was a gift of Dean Edwards, BCM; described in[^4^](#_ENREF_4) |
| (Donkey anti-rabbit IgG-HRP conjugate) | NA934-1ML | GE Healthcare |
| (Sheep anti-mouse IgG-HRP conjugate) | NA931-1ML | GE Healthcare |
| (Rabbit anti-goat IgG-HRP conjugate) | sc-2768 | Santa Cruz Biotechnology |

**Antibodies used for ChIP**

| **Antigen** | **Catalogue Number** | **Supplier** | **μg added** |
| --- | --- | --- | --- |
| KMT2D (MLL4) | HPA035977 | Atlas Antibodies | 3 |
| FLAG epitope | F1804 | Sigma | 5 |
| HA epitope | sc-7392 | Santa Cruz Biotechnology | 5 |
| Normal Rabbit IgG | 12-370 | Millipore | 3 or 5 |
| Normal Mouse IgG | 12-371 | Millipore | 5 |

**Primers and probes used in RT-qPCR Assays**

Roche’s Universal Probe Library Assay Design Center was used for primer-FAM labeled probe synthesis specific to each target gene, which designs primers flanking an intron for use in SYBR green qPCR assays as well. The *GREB1*, *TFF1*, and *ACTB* amplicons have been described[^5^](#_ENREF_5). The table below describes the other amplicons designed in this study.

| **mRNA Assayed** | **Forward Primer (5’->3’)** | **Reverse Primer (5’->3’)** | **Roche Universal Probe** |
| --- | --- | --- | --- |
| *CCND1* | gctgtgcatctacaccgaca | ttgagcttgttcaccaggag | None; SYBR green employed |
| *CTSD* | catcttctccttctacctgagca | gtctgtgccacccagcat | None; SYBR green employed |
| *XBP1* | ccctggttgctgaagagg | tggaggggtgacaactgg | None; SYBR green employed |
| *EGR3* | caatctgtaccccgaggaga | ccgatgtccattacattctctg | None; SYBR green employed |
| *PDZK1* | gacagaattcctgagtgaacga | ggttgaaggtggaggtcattt | None; SYBR green employed |
| *MYC* | caccagcagcgactctga | gatccagactctgaccttttgc | None; SYBR green employed |
| *AREG* | tgatcctcacagctgttgct | tccattctcttgtcgaagtttct | None; SYBR green employed |
| *PGR* | CTGGCATGGTCCTTGGAG | TCATTTGGAACGCCCACT | 89 |
| *ESR1* | ATCCACCTGATGGCCAAG | GCTCCATGCCTTTGTTACTCA | 17 |
| *ESR1-YAP1* | ATGATCAACTGGGCGAAGAG | GCCAAGAGGTGGTCTTGTTC | 58 |
| *KMT2D* | TGAAAGGGCACTGAGGGATA | TGAGGGGGTGTAGGCAAG | 62 |
| *GAPDH* | AGCCACATCGCTCAGACAC | GCCCAATACGACCAAATCC | 60 |

**Primers used in ChIP-qPCR Assays**

| **Amplicon** | **Forward (5’->3’)** | **Reverse (5’->3’)** | **Reference** |
| --- | --- | --- | --- |
| *TFF1* promoter ERE (-0.3 kb from transcription start site (TSS)) | TCACGGCCAAGCCTTTTTCC | CCTCCCGCCAGGGTAAATA | [^6^](#_ENREF_6) |
| *TFF1* ERE3  (-9.9 kb from the TSS) | GTCGTTGCCAGCGTTTCC | CTTCTCCACGCCCTGTAAATTT | [^7^](#_ENREF_7) |
| *GREB1* ERE1  (-1.6 kb from isoform a TSS) | GTGGCAACTGGGTCATTCTGA | CGACCCACAGAAATGAAAAGG | [^8^](#_ENREF_8) |
| *GREB1* putative Enh  (-35.4 kb from isoform a TSS; identified in [^9^](#_ENREF_9)) | CAGGGGCTGACAACTGAAAT | GAGAGGGTGGTGACACTTGG | This study |
| *PGR* Enhancer (-168 kb from TSS) | GATGACAGAAGGAGAAGTTAGAAG | ATATGGCATTGAAGCAACAGG | [^10^](#_ENREF_10) |
| *CCND1* Intron 4 | TGCCACACACCAGTGACTTT | ACAGCCAGAAGCTCCAAAAA | [^11^](#_ENREF_11) |

**siRNAs**

| **Name** | **Catalogue Number** | **Supplier** |
| --- | --- | --- |
| ON-TARGETplus Non-targeting Pool | D-001810-10-05 | Dharmacon |
| ON-TARGETplus SMARTpool NCOA3 | L-003759-00-0005 | Dharmacon |
| siControl-A pool | sc-37007 | Santa Cruz Biotechnology |
| Silencer® Select Negative Control No. 1 (NC#1) | 4390844 | Ambion |
| KMT2C (MLL3) pool | sc-62623 | Santa Cruz Biotechnology |
| Silencer® Select KMT2D (MLL4) | s15605 | Ambion |

**Supplementary Table** **4:** Testing drug synergy between AZD9496 and SI-1 on inhibiting Y537S and D538 ERα-mediated transcriptional activity.

| **Dose AZD (nM)** | **Dose SI-1 (μM)** | **CI (Y537S)** | **CI (D538G)** |
| --- | --- | --- | --- |
| 0.1 | 0.1 | **0.4153** | 3.0618 |
| 0.1 | 0.2 | **0.1791** | 12.6044 |
| 0.1 | 0.3 | 2.9911 | 13.2206 |
| 0.1 | 0.5 | 7.79E+10 | 1.64664 |
| 0.1 | 1.0 | **0.3544** | **0.67967** |
| 0.2 | 0.1 | **0.0667** | 1.85865 |
| 0.2 | 0.2 | **0.4290** | 23.9763 |
| 0.2 | 0.3 | 3.2228 | 24.5925 |
| 0.2 | 0.5 | 1.76E+08 | 1.56427 |
| 0.2 | 1.0 | **0.35441** | **0.68576** |
| 0.5 | 0.1 | 25915.9 | 1.41809 |
| 0.5 | 0.2 | 75366.8 | 2.10384 |
| 0.5 | 0.3 | 2074371 | 3.15145 |
| 0.5 | 0.5 | 6.02E+13 | 1.48451 |
| 0.5 | 1.0 | **0.2774** | **0.65129** |
| 1.0 | 0.1 | 37.2937 | 1.42014 |
| 1.0 | 0.2 | 1581.4 | 2.21168 |
| 1.0 | 0.3 | 19613.5 | 3.46001 |
| 1.0 | 0.5 | 496504 | 1.60281 |
| 1.0 | 1.0 | **0.2774** | **0.66995** |
| 2.0 | 0.1 | 6.8615 | 1.26355 |
| 2.0 | 0.2 | 16.5195 | 1.448 |
| 2.0 | 0.3 | 16.5999 | 1.75928 |
| 2.0 | 0.5 | 74.8526 | 1.30946 |
| 2.0 | 1.0 | **0.2654** | **0.65042** |
| 5.0 | 0.1 | **0.0504** | 1.02752 |
| 5.0 | 0.2 | **0.1047** | 1.35553 |
| 5.0 | 0.3 | **0.2542** | 1.62492 |
| 5.0 | 0.5 | **0.2143** | 1.28113 |
| 5.0 | 1.0 | **0.1420** | **0.55358** |

**CI Value Description:** **< 0.1 Very Strong Synergism**; **0.1–0.3 Strong Synergism**; **0.3–0.7 Synergism**; 0.7–0.85 Moderate Synergism; 0.85–0.90 Slight Synergism

**Supplementary Table** **5:** Testing drug synergy between AZD9496 and SI-1 on reducing cell viability of WT and Y537S ERα- expressing MCF-7 cells.

| **Combo** | **Dose AZD (nM)** | **Dose SI-1 (nM)** | **Total Dose** | **CI (WT)** | **CI (Y537S)** |
| --- | --- | --- | --- | --- | --- |
| 1 | 1.56 | 25 | 26.56 | 5.1073 | 0.9566 |
| 2 | 3.125 | 50 | 53.12 | 1.2717 | 1.265 |
| 3 | 6.25 | 100 | 106.25 | **0.6431** | 0.9991 |
| 4 | 12.5 | 200 | 212.50 | **0.2991** | **0.4794** |
| 5 | 25 | 400 | 425.00 | **0.2676** | **0.3752** |

**CI Value Description:** **< 0.1 Very Strong Synergism**; **0.1–0.3 Strong Synergism**; **0.3–0.7 Synergism**; 0.7–0.85 Moderate Synergism; 0.85–0.90 Slight Synergism

**Supplementary Table 6:** Testing drug synergy between ICI 182,780 and SI-1 on reducing cell viability of WT and Y537S ERα- expressing MCF-7 cells.

| **Combo** | **Dose ICI (nM)** | **Dose SI-1 (nM)** | **Total Dose** | **CI (WT)** | **CI (Y537S)** |
| --- | --- | --- | --- | --- | --- |
| 1 | 1.56 | 25 | 26.56 | 1.6160 | 1.2069 |
| 2 | 3.125 | 50 | 53.12 | **0.8815** | 1.0668 |
| 3 | 6.25 | 100 | 106.25 | **0.7822** | 1.2073 |
| 4 | 12.5 | 200 | 212.50 | **0.7760** | 0.9215 |
| 5 | 25 | 400 | 425.00 | **0.6767** | **0.5697** |

**CI Value Description:** < 0.1 Very Strong Synergism; **0.1–0.3 Strong Synergism**; **0.3–0.7 Synergism**; **0.7–0.85 Moderate Synergism**; **0.85–0.90 Slight Synergism**

**Supplementary Table 7:** List of all bound proteins detected in MS experiments as a searchable Excel file. For each experiment, total number of distinct peptides mapped to gene product (“Peptides”), iBAQ-based fraction of total (FOT) amounts, and amounts corrected for ESR1 amount (“vsESR1”) are shown. “vsESR1” amounts are shown for ESR1 interacting proteins in the main figures, and ESR1 correction and scaling factors are shown in Supplemental Table 2. The first experiment in each dataset served as the control for fold-change estimates. One of the two replicates was chosen as the control, based on where average amount of interacting proteins was higher such that fold-changes are not overrepresented.

**4) Supplementary References**

1 Perissi, V., Jepsen, K., Glass, C. K. & Rosenfeld, M. G. Deconstructing repression: evolving models of co-repressor action. *Nat Rev Genet* **11**, 109-123, doi:10.1038/nrg2736 (2010).

2 Li, S. *et al.* Endocrine-therapy-resistant ESR1 variants revealed by genomic characterization of breast-cancer-derived xenografts. *Cell reports* **4**, 1116-1130, doi:10.1016/j.celrep.2013.08.022 (2013).

3 Cho, Y. W. *et al.* Histone methylation regulator PTIP is required for PPARgamma and C/EBPalpha expression and adipogenesis. *Cell Metab* **10**, 27-39, doi:10.1016/j.cmet.2009.05.010 (2009).

4 Press, M. *et al.* Comparison of different antibodies for detection of progesterone receptor in breast cancer. *Steroids* **67**, 799-813 (2002).

5 Foulds, C. E. *et al.* Research resource: expression profiling reveals unexpected targets and functions of the human steroid receptor RNA activator (SRA) gene. *Mol Endocrinol* **24**, 1090-1105, doi:me.2009-0427 [pii]10.1210/me.2009-0427 (2010).

6 Jaber, B. M., Gao, T., Huang, L., Karmakar, S. & Smith, C. L. The pure estrogen receptor antagonist ICI 182,780 promotes a novel interaction of estrogen receptor-alpha with the 3',5'-cyclic adenosine monophosphate response element-binding protein-binding protein/p300 coactivators. *Mol Endocrinol* **20**, 2695-2710, doi:10.1210/me.2005-0218 (2006).

7 Won Jeong, K., Chodankar, R., Purcell, D. J., Bittencourt, D. & Stallcup, M. R. Gene-specific patterns of coregulator requirements by estrogen receptor-alpha in breast cancer cells. *Mol Endocrinol* **26**, 955-966, doi:10.1210/me.2012-1066 (2012).

8 Foulds, C. E. *et al.* Proteomic analysis of coregulators bound to ERalpha on DNA and nucleosomes reveals coregulator dynamics. *Mol Cell* **51**, 185-199, doi:10.1016/j.molcel.2013.06.007 (2013).

9 Carroll, J. S. *et al.* Genome-wide analysis of estrogen receptor binding sites. *Nat Genet* **38**, 1289-1297, doi:10.1038/ng1901 (2006).

10 Boney-Montoya, J., Ziegler, Y. S., Curtis, C. D., Montoya, J. A. & Nardulli, A. M. Long-range transcriptional control of progesterone receptor gene expression. *Mol Endocrinol* **24**, 346-358, doi:10.1210/me.2009-0429 (2010).

11 Eeckhoute, J., Carroll, J. S., Geistlinger, T. R., Torres-Arzayus, M. I. & Brown, M. A cell-type-specific transcriptional network required for estrogen regulation of cyclin D1 and cell cycle progression in breast cancer. *Genes Dev* **20**, 2513-2526, doi:10.1101/gad.1446006 (2006).
